# Engaging stakeholders in embedded newborn health services/systems research in Kenya; a continuous process involving multiple actors.

**DOI:** 10.3310/nihropenres.13787.1

**Published:** 2024-12-02

**Authors:** Kenneth Karumba, Dorothy Oluoch, Edna Mutua, David Gathara, Sebastian Fuller, Mike English, Fredrick Were, Sassy Molyneux, Michuki Maina, HIGH-Q Stakeholder Representative Group, George Okello, Nelson Muriu, Stephen Kaniaru, Sharon Mweni, Anthony Murage, Daniel Mugendi, Pauline Kamau, Paul Nyamwea

**Affiliations:** 1 Health Systems and Research Ethics, KEMRI-Wellcome Trust Research Programme, Nairobi, Kenya; 2 Faculty of Epidemiology and Population Health, London School of Hygiene & Tropical Medicine, London, UK; 3 Health Systems Collaborative, Nuffield Department of Medicine, University of Oxford, Oxford, England, UK; 4 Kenya Paediatric Research Consortium, Nairobi, Nairobi County, Kenya

**Keywords:** Stakeholder engagement, Newborn unit, Global health, County hospitals, Intervention research moral, strategic and pragmatic components

## Abstract

**Background:**

Engaging relevant stakeholders throughout the research cycle is increasingly recognised as critical to conducting quality health systems research. There are few descriptions and analyses of stakeholder engagement in practice for embedded health systems research especially those that must navigate multi-level decentralised health systems. We describe and reflect on the stakeholder engagement activities of an international multi-disciplinary programme of research focused on newborn care in hospitals in Kenya.

**Methodology:**

Our experienced project stakeholder engagement group coordinated engagement activities across multiple stakeholders with differing interests in the research. We conducted a stakeholder mapping and analysis using an engagement matrix to include national and county-level policymakers, professional communities, associations and regulators, health managers, frontline healthcare workers, patients, families and patient representative groups. Our engagement group maintained a log of engagement activities and had regular programme feedback meetings and discussions. As part of our analysis of stakeholder engagement, we drew on project documents and meeting minutes, and on a conceptual framework which distinguishes between the moral, strategic and practicaldimensions of stakeholder engagement.

**Results:**

Stakeholder engagement was essential but complex in practice. Although we had significant prior engagement experience and relationships at the hospitals and the counties, introducing new staff into NBUs triggered complexities that required careful consultation along the bureaucracies at the counties. Despite the counties having similar hierarchical architectures, engagement processes varied and achieved different outcomes across counties. There were also multiple officeholder transitions over the research period, occasioned by factors in our external environment, often necessitating engaging afresh.

**Conclusion:**

Even with a carefully developed stakeholder engagement plan, an experienced team, and a landscape backed by long-term embeddedness, health research stakeholder engagement can unfold in unexpected ways and requires continuous effort, resources, and adaptability. Meeting the moral, strategic, and practical potential of engagement requires flexibility, responsiveness, and commitment, including adequate resources.

## Plain Language Summary

Talking to relevant stakeholders throughout the research process is getting more recognised as important to conducting exceptional health systems research. Even with this recognition, there is limited practical guidance on how to approach stakeholders, especially in studies involving layered, dynamic autonomous sub-national or local health systems. This paper describes the experiences of our research project focused on newborn care in hospitals in Kenya. The study involved interviews, observations across eight public sector newborn units and an intervention to increase staffing in four of these units.

The Project assembled a team to coordinate engagement activities involving diverse groups, including national and county policymakers, healthcare professionals, hospital staff, patients, and families. The project team adopted a stakeholder mapping approach to ensure inclusion and maintained a log of activities to adapt to emerging challenges. The inquiry into engagement activities was based on project records and a framework addressing the moral, strategic, and practical aspects of engagement.

Engaging stakeholders was key but challenging, especially with the involvement of new project staff getting integrated to work alongside existing hospital staff, which required careful coordination across various levels of the health system. These processes varied between the sub-national governance structures. Engagement was repeated and sustained especially where there were frequent changes in leadership, such as those due to elections. These experiences led us to conclude that despite careful planning and experience, stakeholder engagement in health research often unfolds unpredictably and requires ongoing effort, resources, and adaptability to achieve meaningful outcomes.

## Introduction

Health policy and systems research (HPSR) is a multi-disciplinary and applied field of research aimed at understanding and strengthening the performance of health systems ^1^. Stakeholder engagement has gained prominence in HPSR, particularly for researchers designing, implementing, and evaluating health services interventions and programmes ^2^. Within the health research context, stakeholders are defined as individuals, institutions, institutions, and communities with a ‘direct interest’ or influence in the process and outcomes of the research. According to Deverka, *et al.* (2012), stakeholder engagement is the ‘iterative process of actively soliciting the knowledge, experience, judgment and values of individuals selected to represent a broad range of direct interests in a particular issue” ^3^. Stakeholders in health research typically include community members, health workers, health managers, policymakers, and private sector and non-governmental actors. These stakeholders may be operating at local(micro), sub-national(meso), national(macro) and sometimes international levels.

Stakeholder engagement is often seen as separate and distinct from, but informing, interactions with research participants themselves ^4^. However, a growing set of research approaches build stakeholder engagement into the study design itself, with key stakeholders, and representatives of stakeholder groups and institutions, targeted as key participants ^5^. Depending on the research design and context, the engagement of relevant stakeholders can inform the research throughout the research cycle, from developing the initial research plans and questions, through more detailed proposal development and implementation of the work, to completion of the research and dissemination and support of final research findings. In collaborative, co-designed studies or studies embedded in health systems being examined, stakeholder engagement is woven throughout ^6^. In these ways, stakeholder engagement is integral to the anticipated pathways to impact ^7^. Failure to appropriately engage relevant stakeholders potentially undermines the quality of learning, and interest in the study findings, and – given that stakeholders include those expected to hear about and act upon study findings - ultimately the impact of the research on policy and practice ^4, 8^.

Despite the emphasis on stakeholder engagement in the HPSR literature, there are few descriptions and analyses of engagement in practice for different study designs ^9^. The available literature suggests that stakeholder engagement is often skewed towards setting up studies ^10^ and sharing study findings ^11^ with little attention to some of the complexities and tensions in managing and responding to stakeholder inputs throughout the research process and (where relevant) to study team withdrawal from facilities. Additionally, there is a dearth of ‘thick’ descriptions of stakeholder engagement across layered institutional arrangements such as from the hospital level to higher levels of bureaucratic structures in a devolved government.

Past work by ourselves and our colleagues has highlighted the importance of purposefully selecting stakeholders to fit project needs, and of clearly defining the roles and expectations of researchers and other stakeholders from the onset. We have emphasised the importance of involving ‘across-system’ actors including those often overlooked in such engagement activities (such as frontline health workers) and recognising and responding to the dynamic nature of stakeholder’s involvement over a project’s lifetime ^1, 12– 16^ Engagement activities have combined those that are more and less interactive, across the continuum of engagement from consultation to collaboration. The International Association for Public Participation distinguishes between ‘inform’, ‘consult,’ ‘involve,’ ‘collaborate’, and ‘empower’ ^17^ with increasing depth of engagement across these forms of engagement. This categorization has aided in planning, implementing, and evaluating stakeholder engagement and communication.

In this paper, we describe and reflect upon stakeholder engagement for a multi-disciplinary collaborative HPSR study – the ‘Learning to Harness Innovation in Global Health for Quality Care (HIGH-Q) programme – which was conducted in the layered public health system of Kenya. This Programme is seeking to build on our relationships with stakeholders and lessons from past engagement in related studies. Specifically, in this paper, we provide an overview of the project stakeholder engagement strategy, describe the varied engagement activities and outcomes from devolved units with similar structures and mandates, as well as discuss the implications for policy and practice for similar studies in the future.

## Study setting

### The HIGH-Q Programme 2020–2025

The HIGH-Q programme, implemented in Kenya, is a partnership between the Kenya Medical Research Institute (KEMRI)-Wellcome Trust research programme, the University of Oxford, the Kenya Paediatric Research Consortium, the London School of Hygiene & Tropical Medicine and the County Departments of Health. Running from October 2020 to September 2025, the HIGH-Q Programme of work comprises a co-designed, multi-disciplinary intervention and evaluation to study the effects on nurse-delivered care of a prospectively designed workforce intervention aimed at improving nurse staffing in select neonatal units in Kenya. The workforce intervention was built around and concurrent with a multi-country intervention programme known as Newborn Essential Solutions and Technologies (NEST360) ^18^. The NEST360 technology intervention is implemented in a large subset of a network of county hospitals known as the Clinical Information Network (CIN) (See Box 1 for a summary of NEST360 and CIN); the HIGH-Q programme involved a smaller subset of these NEST360 sites.

**Box 1. The Programmes linked to HIGH-Q**

**NEST360** comprises a global coalition of clinical, biomedical, and public health specialists hailing from 22 prominent institutions and organizations. The alliance's main objective is to assist African governments in implementing a comprehensive care package encompassing cost-effective technologies, training programs for both clinicians and biomedical technicians, and locally managed data systems, all aimed at ensuring the delivery of high-quality care for small and sick newborns ^18^.

**The CIN** is housed at the Health Services unit of the KEMRI-Wellcome Trust. Operating since 2013, the CIN is a collaboration involving the national Ministry of Health and the departments of health at the county governments. The Network has expanded to include 24 hospitals, as well as policymakers and researchers, with an aim to develop and adopt evidence-based clinical guidelines for paediatric care, enhanced research feedback to hospitals and as a vehicle for learning health systems focused on paediatric and neonatal care ^12, 16^.

The HIGH-Q programme has a specific focus on neonatal care in Kenyan hospitals. The programme is assessing how introducing additional workforce alongside the NEST360 technologies in Kenyan hospitals affects technology adoption and quality of care in light of known and substantial workforce deficits ^14^. Completed in July 2023, the specific workforce intervention aimed to increase nursing and ward assistant numbers in newborn units ^19^. Specifically, the intervention involved the employment of three additional nurses and three ward assistants in each of the four-county hospital newborn units for 15 and 7 months respectively. Salaries for these staff were paid by the HIGH-Q programme for the research period, but the staff were employed and line-managed by the hospitals. The other HIGH-Q objectives examine health workers and mothers’ experiences of newborn care, how to better support the delivery and integration of post-discharge care for families, the governance process of introducing technologies and service delivery innovations and infection prevention control in these newborn units. Primary data collection includes structured and unstructured observations of care and technology use and interviews with healthcare professionals (at all levels of the health system) and carers. Table 1 summarises HIGH-Q Programme work packages, research questions and methods.

**Table 1.** HIGH-Q Programme Work packages, research questions and methods.

**WP1a: Health workforce intervention and indicators of quality of newborn care** **Question:** If staff-to-patient ratios are increased by adding new staff in NBUs does this improve quality of care?		
Methods	Sites	Sample(accomplished)
Direct observation of care using nursing care index tool, tracking workforce changes using staff rota	4 intervention county hospitals 4 Non-intervention County hospitals	Babies observed= **1,302** (948 at intervention and 354 at non-intervention sites), **10,379** hours of observation in intervention sites (+3,938 hours at Non-intervention sites)
**WP1b: A process of delivering and implementing technologies and uptake factors** **Question:** When new technologies are introduced in NBUs what effects does this have on staff and families?		
Methods	Sites	Sample(accomplished)
Observation, informal and formal interviews with health workers and mothers as well as document review	4 intervention county hospitals	Mothers interview **n=38,** Nurses Interviews **n=101** House of observation **n=3500**
**2A: The process of post-discharge neonatal care and how innovations can improve pathways to care** **Question:** * How can we work with hospitals, staff and families co-design better post-discharge care services for at risk newborn and their families?*		
Methods	Site	Sample(accomplished)
Observation, informal and formal interviews with health workers and mothers as well as document review	4 intervention county hospitals	4 co-design workshops, 15 interviews with healthcare providers, 21 interviews with mothers. Observation (160 hours in hospitals, 34 hours in homes)
**Wp3: The governance process of introducing technologies and service delivery innovations** **Question:** *How do global, national, and local governance structures and processes interact to shape the introduction and oversight of * *new and existing medical devices in Kenyan hospitals?*		
Methods	Site	Sample(accomplished)
Informal and formal interviews with health workers and health, regulatory and NEST 360 managers and document review	4 intervention county hospitals, 1 Non-intervention County Hospital, 2 other additional hospitals.	32 interviews with health workers and NEST 360 staff. 3 FGDs with health workers, 3 interview with national managers, Informal discussions with national and county stakeholders

In Kenya, health care is devolved from the national government to 47 semi-autonomous sub-national governments (commonly known as counties) ^20^. Under this governance arrangement, county governments have an executive, administrative and legislative mandate. From the early study planning stages for HIGH-Q, it was recognised that stakeholder engagement was needed at national, county, hospital and newborn unit levels, and should build upon and consider wider CIN and NEST360 activities, as well as any other research that might be ongoing at these sites during this period. 

## Methods

An experienced programme stakeholder engagement group was established at the outset of HIGH-Q to coordinate stakeholder engagement. The stakeholder engagement group constitute the Programme’s principal investigators and researchers, the majority of whom are from or have lived and worked in the Kenyan health system for over a decade. The KEMRI-based research group is coordinated by a project manager, the first author (KK), who himself has health system management experience and masters-level training in public administration.

Stakeholder engagement activities were integrated at the various stages of the Programme from proposal development to pre-intervention period and during the various phases of the Programme’s implementation.

An initial engagement strategy consisting of the goals for engaging stakeholders was developed at the proposal development stage This strategy adopted a Rainbow diagram for stakeholder identification adapted from Chevalier and Buckles (2008) ^21^. Under this model, stakeholders were identified stakeholders and placed in concentric layers depending on influence, interest and how close they were to the NEST360 and the HIGH-Q research. Identified stakeholders were categorized from layered perspective from those closer to the research to those further away. The process yielded multiple stakeholders who included the national and county level policymakers, medical and nursing professional communities, professional association regulators, health managers at various levels of the health system, frontline healthcare workers in hospitals, and hospital patients, family members and patient representative groups. These stakeholders especially those close to the research were engaged throughout the HIGH-Q research process.

After the mapping exercise, an engagement matrix analysis was conducted to understand who the stakeholders in the planned research are, their composition, their interests and concerns, and how they are likely to influence the study's success. The matrix allowed for the articulation of the engagement activities, key messages, any necessary materials and timelines. Stakeholders were reached through official letters signed by the principal investigator and posted via courier. Follow-ups of the official correspondences were done through email and phone contacts retrieved from a CIN contacts list shared across projects in the network working with similar hospitals. Key messages were crafted based on specific aims of stakeholder engagement encompassing ‘inform’, ‘consult,’ ‘involve,’ ‘collaborate’, and ‘empower’ as advanced by the International Association for Public Participation ^17^.

Stakeholder engagement meetings were held in person in various national and county offices, in conference meeting rooms and in hospitals, complemented by follow-up online meetings, telephone calls, email correspondence, posted mail and a newsletter. Feedback on stakeholder priorities, concerns and complex scenarios emerging from the engagement was relayed to the principal investigators and the wider scientific team through regular meetings and individually, as needs arose. 

The entire engagement process was carefully documented and tracked in a log/diary by the project manager. To write this paper, we drew on recorded activities and meeting minutes gathered between October 2021 to February 2024, a period covering the Programme’s proposal development, introducing and conducting the main study fieldwork, and beginning to feed back the early findings from the research.

### Synthesis and Interpretation of Learnings

To support our reflections of the impact of the Programme’s stakeholders’ activities, we have applied ideas from Kujala, Sachs, Leinonen, Heikkinen & Laude (2022) conceptual framework which proposes engagement activities to consist of moral, strategic, and pragmatic components ^22^. Their framework is a response to what is observed by the paper as a fragmented approach to analysing stakeholder engagement which hampers research progress. The **moral component** involves considering the ethical dimensions of how the operations of an organisation affect individuals and groups rather than focusing on organizational interests. In the context of our research, we consider for example how to engage particularly marginalized stakeholders, how to ensure respectful interactions with all stakeholders, and how to ensure the research is made meaningful to diverse stakeholders. The **strategic component** is concerned with how managers distribute scarce resources among stakeholders in ways that ensure value creation for the organisation and the achievement of its objectives. In the context of our research for example we recognise that we have deliverables to ourselves and our funders with regards to quality of research and our contribution to knowledge. The **pragmatic component** could be seen as working across the moral and strategic components. Drawing on Chinyio and Akintoye (2008) ^23^ and Forsythe, Ellis, Edmundson *et al.* (2015) ^9^, we considered these as pragmatic activities to build and sustain connections. Including employing negotiations, making trade-offs and adjusting to the practical requirements of stakeholders.

Under this approach to reflection stakeholder engagement in the programme were assessed if they met the three goals of engagement of moral, strategic and pragmatic by analysing the element of the of Programme specific activities against the three components. 

## Results

We begin by describing our initial strategic plan for the engagement, followed by sharing some of our experiences in navigating interactions across a diverse and evolving stakeholder landscape. We also outline the outcomes of our engagement across counties. Finally, we outline our approach to the dissemination of research findings and exit strategies after project completion.

### Developing an initial strategic plan to guide stakeholder engagement

**Figure 1.**
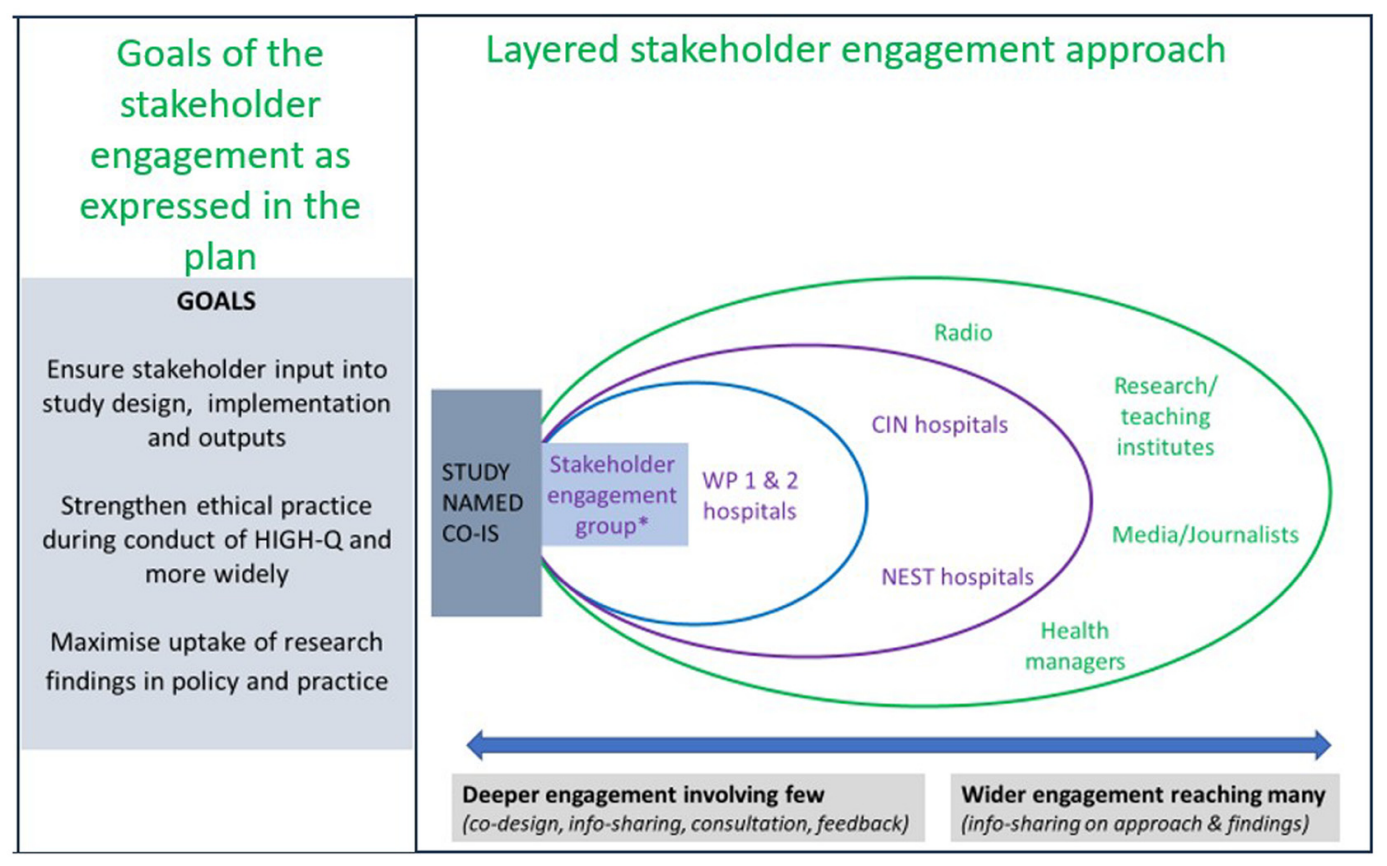
The HIGH-Q Community and Stakeholders’ engagement framework goals and the components of engagements.

During the proposal development and the programme’s setup phases, the investigators formulated an initial framework for engaging stakeholders. This framework encompassed engagement objectives, detailed stakeholder mapping, and the nature and content of engagement activities ( Figure 1). The overall inter-related goals of stakeholder engagement were to contribute to 1) good quality science through inputs into study design, implementation and outputs; 2) ensuring ethical practice throughout the research process, and 3) maximising the uptake of research findings in policy and practice.

The engagement framework covered a full spectrum of activities. At one end of the spectrum was deep engagement involving fewer people in more interactive ways. Activities include engaging stakeholders in consultative meetings to co-design elements of the research and get advice on the stakeholder engagement plan itself, as well as on how to conduct the research. At the other end of the spectrum are wider engagement activities aimed at reaching many people through inevitably less interactive activities. Examples include radio programmes, video outputs, newspaper articles and policy briefs. 

At the heart of the engagement activities, and to advise on all activities, we set up international and national advisory expert groups with diverse types of expertise. The international expert group includes seven stakeholders with global health research experience and expertise in medical sociology, health economics, organisational analysis, health workforce and systems, infectious disease epidemiology, regional nursing policy and advocacy for nursing and maternal and child health. The national expert group consists of eight individuals with representation from the national Ministry of Health, medical schools, medical and nursing professional communities, professional association regulators, and patient representative groups. These groups were consulted together at least annually supplemented by periodic interactions with members as needed.

### Implementing engagement across a complex and expanding range of stakeholders

**Figure 2.**
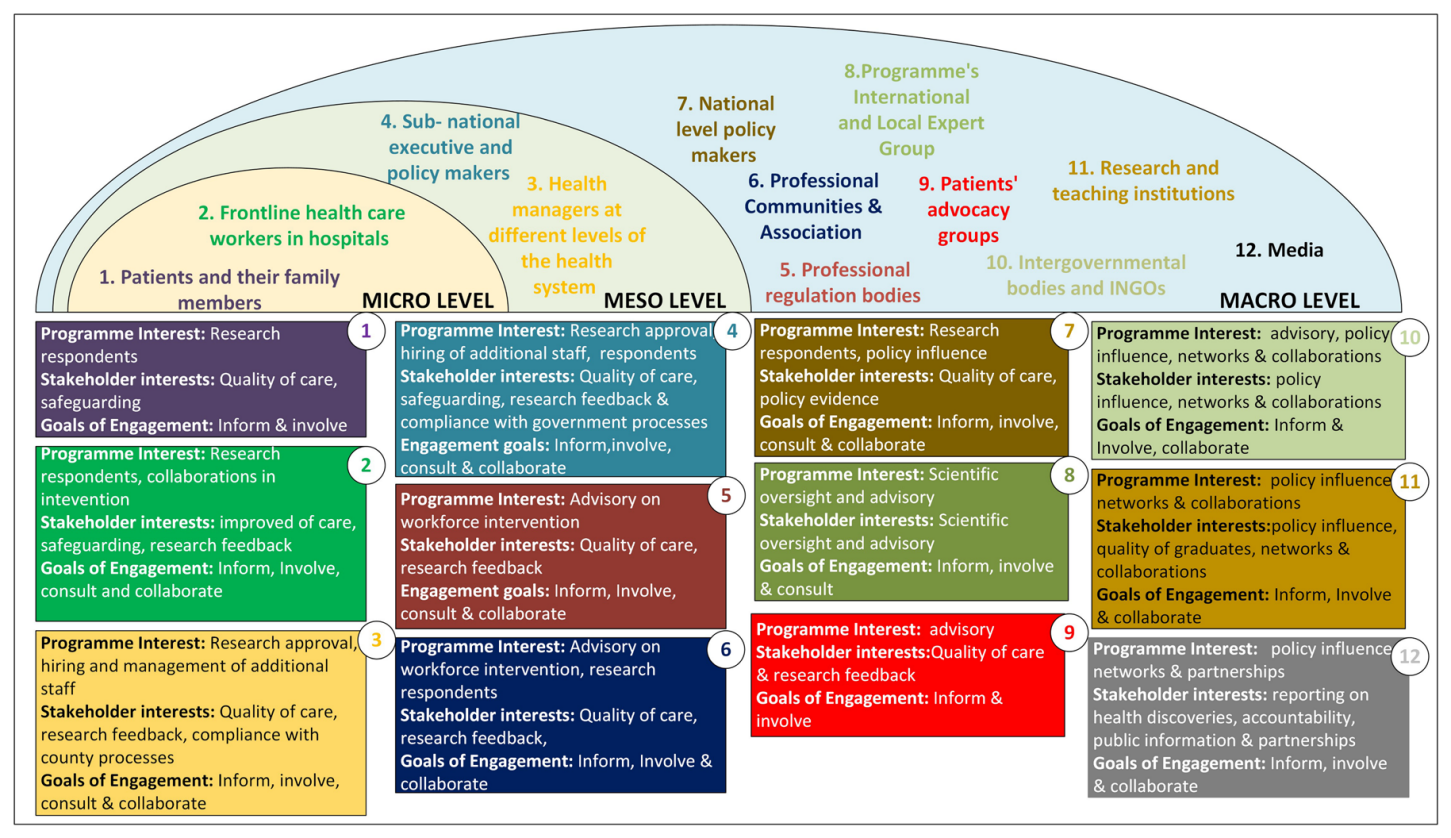
Presentation of multiple stakeholders engaged each with different interests.

Across the activities, we engaged a wide range of stakeholders based on our understanding of their needs, interests and concerns ( Figure 2). Drawing on the International Association for Public Participation distinctions described above, we ****informed**** all stakeholders about key information to help them comprehend the research issues and support further engagement. We also ****involved**** representatives of all stakeholders (including research participants), to ensure their concerns were understood and considered in research activities and study learning. We ****consulted**** with representatives of many stakeholder groups to gather input regarding our plans, including through our advisory groups and had formal ****collaborations**** with stakeholder groups drawn from the counties and hospitals. We sought to ensure the research was as responsive as possible to stakeholders’ needs and priorities and would not have been able to proceed if gatekeepers such as regulators and county and hospital managers had not approved the study. Nevertheless, the final decision-making on the study details remained with the study team. In these ways, we were continuously balancing ethical, strategic and pragmatic elements of stakeholder engagement. For the workforce intervention level, the recruitment approach followed county procedures as described in a later section. At the NBU level, nurse managers were fully empowered to manage the duties and performance of additional nursing staff.

**Figure 3.**
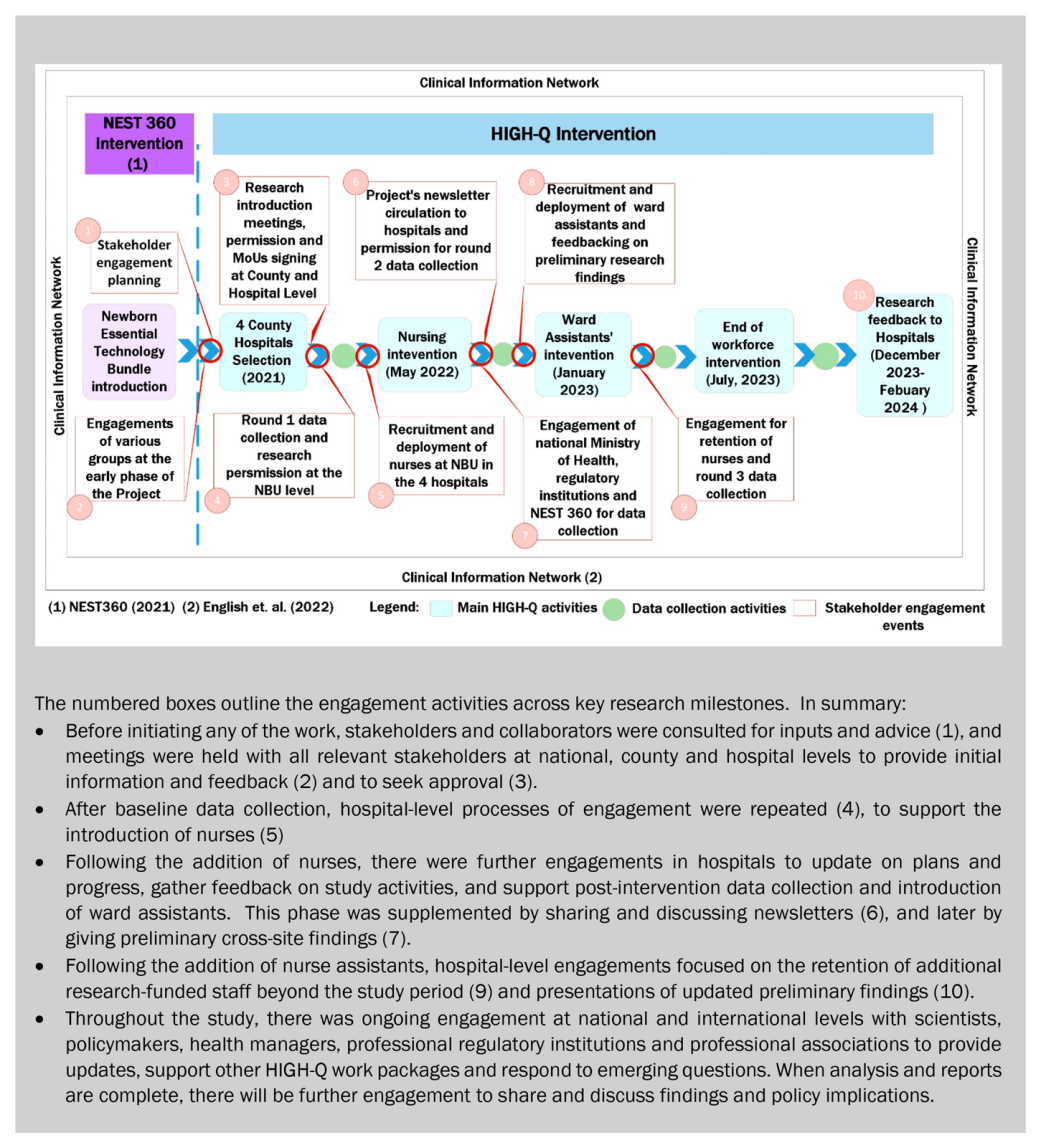
A summary of the HIGH-Q Community and Stakeholders’ engagements against Programme milestones.

As presented in Figure 3, there were three main observations from this work. First, the bulk and the backbone of stakeholder engagement focused on the four hospitals, especially on assessing the technology introduction and workforce enhancement effects on care, staff, and families. We were aware that the engagement of leadership and health workers is critically important and had planned an initial engagement with the county director of health followed by engagements down through the hospital hierarchy to newborn units. However, the workforce component significantly expanded the complexity of our engagement processes as outlined further in the following section.

Our second observation is that we had to respond to field questions and realities with many informal or unplanned follow-up engagements or activities, in response to issues raised by the study teams or stakeholders. Examples include:

- Ward staff raised informally that there were too many research staff conducting observations or interviews in a facility at one time, potentially impacting data collection and patient care (co-authors EM, DO, SF, MM, SM) are assessing this concern and other observed ethical issues arising from conducting the HIGH-Q research). In response, HIGH-Q fieldwork was reorganized, and new ways of working were shared back with hospital stakeholders.
- Hospital managers mentioned that overlaps and links between NEST360, CIN and HIGH-Q were unclear, potentially leading to confusion. In response, communications were re-organised and rewritten to be more coordinated and to clarify differences and support referral to other groups as needed.

We observed that while some of these issues could be considered primarily practical, strategic, or moral, there were often interactions and interplays between the two. For example, having too many research staff on a ward had practical implications (difficult to collect data) and moral implications (disruption to routines of busy, often overwhelmed staff and to care of vulnerable children). Our third observation was that some stakeholder groups were more challenging to engage in terms of reaching them and achieving sustained involvement than others. For instance, our engagement with parents was primarily through interviews and observations, preceded by consent, and informal interactions. Linked to research governance, our study was subject to review and approval by the National Commission for Science, Technology, and Innovation (NACOSTI) and our international researchers were required to apply for and obtain research permits from the Immigration Department. Still, on research governance, we had quite a bit more interaction with nursing regulators / professional bodies such as the Nursing Council of Kenya (NCK) and the National Nursing Association of Kenya (NNAK). Our engagement with these regulatory institutions involved following all requirements at international, national, and local levels for initial approval and where necessary annual reports and renewal. At the data collection level, we reached out to a range of regulators of health research and technology to conduct interviews, with varying success.

As part of reaching out to an even wider audience, we plan to engage with teaching institutions and the media as part of the next phase of stakeholder engagement.

### Different approaches and outcomes between counties with similar architecture

**Figure 4.**
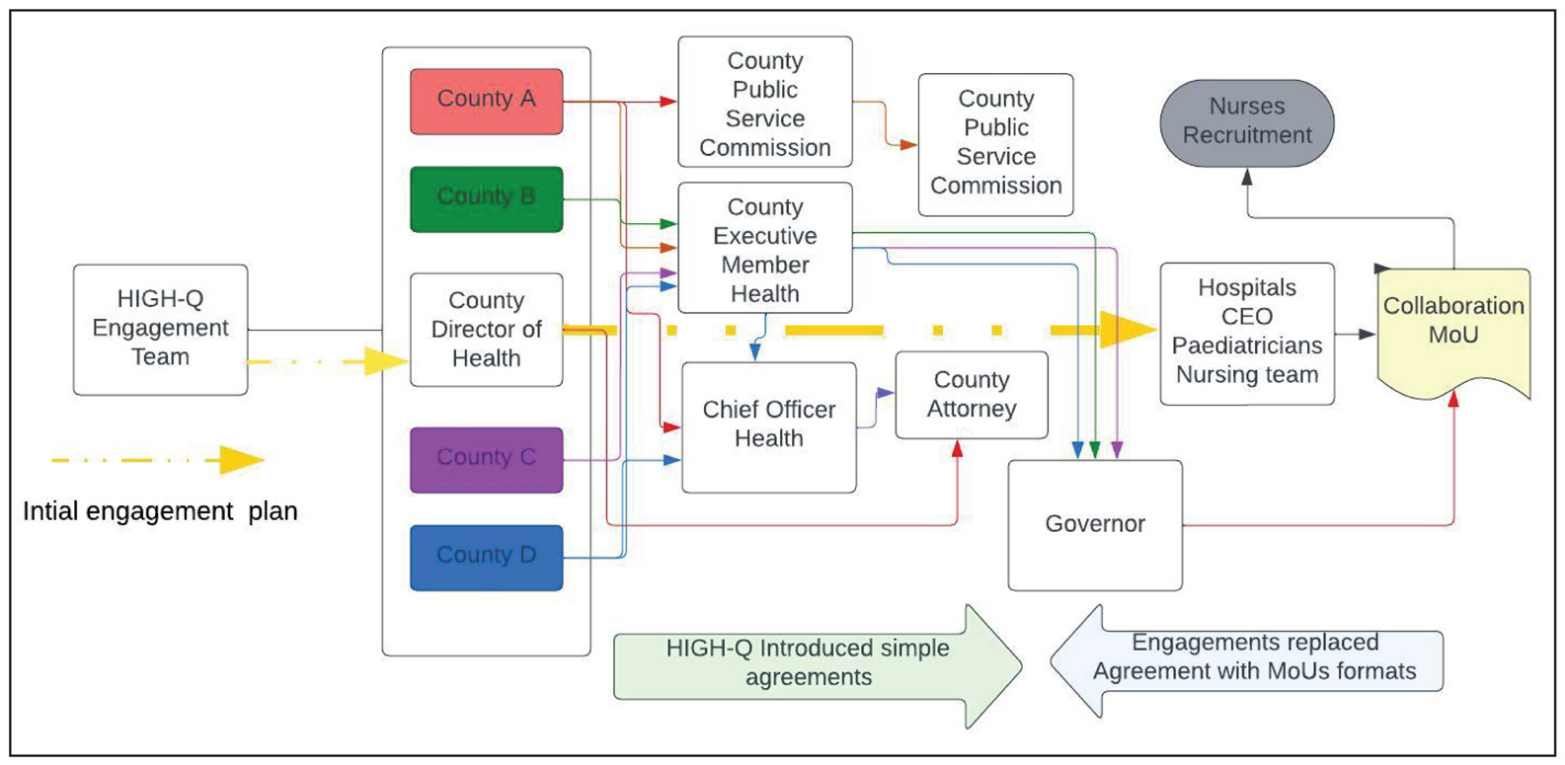
A presentation of County and Hospital structure and the evolving engagement by the HIGH-Q Programme.

In the county structure in Kenya, there were several important officeholders to consider in any hospital-based engagements (See Box 2 on County Governments and the Departments of Health structure relevant to the study, adapted from the County Government Act, 2012 ^24^ and the Transition to Devolved Government Act, 2012 ^25^). In the HIGH-Q programme, the nature of the workforce intervention - with additional staff employed and managed by the county government - triggered a more complex set of engagements than is typically required for more observational or descriptive research. Figure 4 illustrates our original linear simple partnership plans in the centre. These morphed into many referral contacts, many people to talk to and get permission from and eventually the conversion of simple agreements into complex Memoranda of Understanding (MoU). To get permission and approval for this aspect of the research, we had to engage with chief officers, the county executive committee member, a county public service board, the county legal offices, the county secretary, and the governors. These engagements differed across counties, leading to differences in the ways additional staff were employed, despite the similarity in county hierarchical and administrative architecture.

**Box 2. County Government and Departments of Health structure relevant to the study**

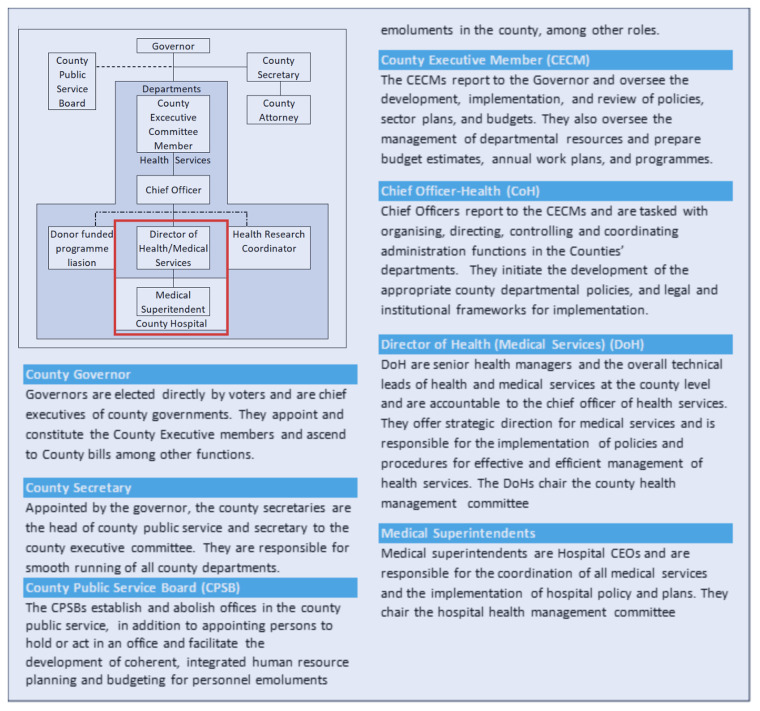

Other notable differences were in the engagement frequency and duration in the four counties to gain approvals and on other research-related events. Table 2 below presents quantitative data on the frequency and duration of engagement with different county officials in the pre-intervention (PRE I) period comprising 7 months, and during the intervention (DUR I) comprising 15 months. The pre-intervention period was before the MoU signing and the recruitment and deployment of additional staff. The intervention period consisted of deploying staff working in the units and data collection. The number of engagements in this period with county and hospital-level senior office holders ranged from 5 (County A) to 23 (County D). The combination of methods employed for engagement consisted of face-to-face, phone calls, email correspondence and letters. A majority of county and hospital executives were engaged directly and at varying durations (presented numerically in brackets and consisting of weeks of engagement from initial contact to sharing of findings) and frequencies (also presented numerically). Those closer to the intervention (the medical superintendents) were engaged multiple times, while those further from the intervention (the governors and the CECMs) less often.

**Table 2.** A presentation of Frequency and Duration of engagements- pre- and during intervention.

County and Hospital persons engaged	Duration- No. of frequency (weeks)							
	County A		County B		County C		County D	
	PRE I	DUR I	PRE I	DUR I	PRE I	DUR I	PRE I	DUR I
Governor	N/A	N/A	1 (6) *	N/A	1(3)	N/A	1 (2)	N/A
County Secretary	1 (5)	N/A	N/A	N/A	N/A	N/A	N/A	N/A
County Public Service Board	14(30)	12(8)	N/A	N/A	N/A	N/A	N/A	N/A
County Executive Committee Member Health	1(1)	2 (3)	1(6)	N/A	3(10)	2(3)	(3)5	2(3)
County Legal Officer	N/A	N/A	1(1) *	N/A	3 (3) *	N/A	N/A	N/A
Chief Officer Health	4(3)	1(1)	N/A	N/A	N/A	N/A	N/A	N/A
County Director of Medical Services	22(12)	N/A	46 (30)	7(65)	12(30)	(17)65	N/A	N/A
Medical Superintendent	4(30)	5(65)	16(30)	23(65)	7 (30)	8(65)	17(30)	11(65)

*Engaged indirectly

The differences in engagement pathways between counties were reflected in MoU execution and recruitment of staff ( Table 3). We assessed MoU attributes according to the number of signatories, number of articles, and weeks from initiation to full execution. We see that County A added an extra clause on recruitment by the County Public Service Board (CPSB), and County B was directly coordinated by the county director of health (DoH) who worked closely with the County’s legal team to develop and sign a complex MoU with multiple clauses. The process at County B took double the time of Counties A and C, where the process was coordinated by the HIGH-Q team.

**Table 3.** A presentation of the dynamics of MoU execution experiences in the four counties.

	County	County A	County B	County C	County D
**MoU ** **Attributes**	**Highest ranking ** **signatory**	County Secretary	Governor	CECM	COH
	**No. of County ** **Signatories**	5	2	3	2
	**No. Articles in ** **MoU**	8	23	7	7
	**Duration to full ** **execution (weeks)**	7	14	7	9
**Recruitment ** **of staff**	**Approaches to ** **Recruitment of ** **Nurses**	Through CPSB	Through the Programme with the Director of Health represented in the interview *	Through the Programme with the Director of Health represented in the interview	Through the Programme with the medical superintendent represented in the interview
	**Recruitment of ** **Ward assistants**	Through CPSB	Through the Programme with candidates getting nominated by hospital	Through the Programme with candidates getting nominated by hospital	Through the Programme with candidates getting nominated by hospital

* County B deployed recruited staff in other department and utilized existing staff for the intervention

Our experiences underscored the importance of being able to be flexible and responsive in our engagement and study plans. Of note is that the entire process above was also complicated by routine job (re)postings and by national general elections. At the governor level, three of four governors and all the four Executive Committee members (CECMs) at the Department of Health in the four counties exited after the general elections, and in one of the counties, there were 3 different CECMs during the engagement period. All four Chief Officers at the Department of Health (COHs) left after the elections, although the County Directors of Health remained, and all hospitals registered a transition in the office of the medical superintendents during the engagement period. The first transition of medical superintendents happened only one month after the commencement of engagement at County C and the last a month before the end of the engagement period. These changes led to the need to regularly engage afresh, especially where the new office holders were closely involved in the implementation, such as medical superintendents. In County A&B the transition happened before the MOUs had even been signed.

### Engagement for research feedback and study withdrawal

As illustrated in Figure 3, we undertook two cycles of research feedback at the four county hospitals during the engagement period, with further feedback and discussion of findings planned over the next 6–12 months.

The first research feedback meeting took place between November and December 2022, six months into the nursing workforce intervention and just before the introduction of ward assistants. The objective of this feedback was to give progress updates and preliminary research findings from the baseline survey. The findings were anonymized across all the four sites. The audience targeted included the medical superintendent, paediatrician, nursing leaders and various departmental staff. The feedback provided further opportunities for hospitals to understand the qualitative work and consenting process, and to promote discussion on aspects of care the hospitals were eager to improve. Stakeholders expressed being keen in future to have hospital-specific feedback.

The second set of feedback was given in December 2023 and February 2024, a year later, after completion of all the fieldwork, and following further analysis. Before feedback in each hospital, the research team spent considerable time preparing feedback data presentations and crafting appropriate messages to hospitals. Hospital-specific data were provided with comparative anonymized data from other hospitals. The NBU hospital teams were interested in discussing the clinical data but were also concerned about the withdrawal of intervention staff once the study was over.

In response to NBU team requests, and as part of the withdrawal plan from hospitals, we conducted an additional extensive set of engagements with county stakeholders at the departments of health in all four counties to explore the retention and absorption of intervention staff into the county workforce. We corresponded with all county and hospital executives previously involved in the study approval process - county secretary (County A) CECMS (all counties) CoH (County A &C) DoH (all counties) and medical superintendents (all counties). Our engagement involved a brief highlight of existing partnership and contractual arrangements for the HIGH-Q programme, an update on the status of workforce enhancement and the short-term employment of intervention staff, an assessment of the contribution of the additional workforce to the quality of newborn care at the NBUs and finally to explore opportunities to retain and absorb the project nurses. These efforts culminated in a brief face-to-face meeting with three of the four CECMs at a CECM forum. The feedback from the CECMs cited county HR practices which promote a competitive recruitment process, leaving no room for the direct absorption of staff from the Programme into the county public service. Options for inviting the HIGH-Q Programme nurses to be part of recruitment once public funding for staffing at the county was secured were discussed, offering some promise that staff would be afforded opportunities through open recruitment. This discussion, however, took place at a time of a major problem nationwide in recruiting new health staff linked to national budget caps with direct implications for the County governments. The Public Finance Management (National Government) Regulations, 2015 (Kenya) ^26^ imposes a limit of 35 per cent expenditure on county staff salaries and benefits against total county revenues. According to the latest report available, on average, the County governments have exceeded this limit ^27^.

## Discussion

Involving key stakeholders at every stage of the research process is now widely acknowledged as essential for producing high-quality, impactful health policy and systems research. For the HIGH-Q programme, the study design and past experiences have contributed to extensive engagement with multiple international and local stakeholders. Stakeholder engagement has gone far beyond access and compliance requirements, to embrace ethical principles such as ensuring respectful interactions seeking to maximise benefits and minimise inconvenience to all involved. Stakeholder engagement was essential to being able to conduct the study.

In engaging multiple stakeholders, we found the International Association for Public Participation distinctions between ‘inform’, ‘consult’, ‘involve’, ‘collaborative’, and ‘empower’, valuable in helping plan our engagement work, specifically in refining objectives for engagement and in designing communications and agendas for specific stakeholders. As observed by Potthoff *et al.* (2023) the classification helped us develop some level of conceptual clarity for our activities, which were also guided by our engagement goals and past work ^28^.

Each of the stakeholder engagement goals ( Figure 1), and many of the engagement activities ( Figure 2) and ( Figure 3) have moral, strategic and pragmatic elements. For example, the engagement of international and national experts could be viewed as strategic in ensuring that good quality research is planned and conducted. However, helping ensure that the research is context-relevant, potentially impactful and conducted in ways that are sensitive to work environments and cultures are also morally relevant and comprised of pragmatic inputs. Similarly, the engagement of counties and hospital senior staff for initial approval and later to give study feedback and advocate for the retention of intervention staff had elements of all three components.

The potential value of distinguishing between these elements in a context like our research could be in the research team regularly reflecting on the balance across the three components, and whether this remains appropriate across the shifting lifespan of a programme.

Prebanic and Vukomanovic (2023) observed that the engagement of project stakeholders can be highly complex because of stakeholders’ conflicting interests which can lead to time and cost overruns ^29^. In our experience, complexity arose less from conflicting interests, than from the need to seek buy-in and inputs from a large and expanding set of stakeholders at different levels of the health system hierarchy, and to having to continuously re-engage with stakeholders as the study progressed. This engagement was aimed at maximising stakeholder input and interest and, in turn, aimed at strengthening the potential for uptake of study learning into policy and practice discussions during the study and after its completion. In these ways, we were seeking to balance the strategic, moral, and practical elements of engagement as our work and the contexts evolved and shifted.

Our engagement needs were intensified by being centred on a workforce intervention with the new staff operating as part of normal hospital duties with output contributing directly to hospital service delivery. In Kenya, a trend of ‘recentralization within decentralization’ has been described by Barasa *et al.* (2017), whereby hospitals have lost some of their pre-devolution autonomy to county level departments of health higher up the health system hierarchy ^30^. For the HR function, part of hospital autonomy was surrendered to the county public service board during the devolution. For these reasons, it was essential to engage county Departments of Health -and in specific instances, the county public service board, the county secretary, and the governor before additional ‘project staff’ were employed. Although we may have simplified the human resource management process, and our engagement activities, if we had opted to directly contract and manage the project staff payroll, failure to follow the prescribed recruitment path would have undermined the study objectives as well as the uptake of research findings.

Murphy *et al.* (2021) has described the importance of acquiring an exhaustive contextual understanding before engaging in the implementation of a global health project ^31^. Adhikari, *et al.* (2019) has highlighted the importance of working through local hierarchies to seek permission for research ^32^. Our findings support and extend these arguments. Our long-term, embedded relationships supported a rich contextual understanding, but this learning continued to evolve throughout our work, highlighting the importance of a well-resourced, continuous, and responsive stakeholder engagement process. Particularly important to responsiveness was the study team’s willingness and ability to respond to stakeholders' formal and informal requests for information. Regarding working through hierarchies, planners of multi-site programmes like HIGH-Q may assume the process of engagement to be the same where established sub-national and local structures are similar. Our findings on the variation of time taken, the structure of the MoUs, the numbers of individuals engaged and the route of the engagement in the governance hierarchy demonstrated that this may not be the case.

Even with a well-established long-standing relationship like the CIN network and freshly instituted relations like those of the HIGH-Q programme, transitions occasioned by high turnover of public sector bureaucrats make established relationships, and thus stakeholder buy-in transient ^32^. For the HIGH-Q programme, this transition was largely due to general elections, which happened one year into the implementation, as well as from more routine postings. These local-level transitions remind practitioners and project designers of the need to pay attention to the ever-changing contextual landscape and transactions such as those that may impact the movement of the population, especially for longitudinal studies.

## Conclusion

Stakeholder engagement has gained prominence in HPSR among researchers and funders with its integration going beyond project kick-off to implementation, project close-outs and knowledge translation. Engagement can be aided by elaborate stakeholder engagement plans, executed by experienced engagement teams incorporating learning from frameworks and contextual experience. However, the process is often complex and unpredictable, shaped by hierarchical structures, bureaucratic requirements and shifting power relations. In addition, the process has its costs in time and money, we were fortunate to have funding support from the UK’s National Institute of Health and Care Research (NIHR). We have shared experiences in managing engagements with multiple stakeholders and the triggers of engagement complexities. Meeting the moral, strategic, and practical potential of engagement requires flexibility, responsiveness, continuous efforts and commitment, including adequate resources.

## Ethics approval

Ethical approval for the study was obtained from the KEMRI Scientific and Ethics Review Unit, Ref: KEMRI/SERU/CGMR-C/229/4203, dated 27 May 2021, with annual renewals on 13 May 2022 and 18 May 2023. Approvals were also received from the County Departments of Health of the four participating intervention hospitals.

## Consent

This report is based on the description of stakeholder engagement process in newborn health services by the programme stakeholder engagement team rather than a research study. As such, no consent was required to analyse programme documents used in the study.

## Data Availability

Data used in this report is protected under national laws and institutional policies and clauses in the MoUs and therefore restricted. Due to ethical institutional and legal restrictions, supporting data can be available on request to the corresponding author and upon review and institutional approvals.

## Appendix 1: Members of the HIGH-Q Stakeholder Representative Group

- Dr George Okello, Country Director, NEST360 Kenya
- Dr Nelson Muriu, Department of Health Services, County, County Government of Nyeri
- Dr Stephen Kaniaru, Department of Health Services, Embu County Government
- Dr Sharon Mweni, Department of Health Services, Machakos County Government
- Dr Anthony Murage, Department of Health Services, Kiambu County Government
- Dr Daniel Mugendi, Department of Health Services, Embu County Government
- Dr Pauline Kamau, Department of Health Services, County Government of Nyeri
- Dr Paul Nyamwea, Department of Health Services, Machakos County Government
