# Engaging stakeholders in embedded newborn health services/systems research in Kenya : continuous process involving multiple actors

**DOI:** 10.3310/nihropenres.13787.3

**Published:** 2026-06-09

**Authors:** Kenneth Karumba, Dorothy Oluoch, Edna Mutua, David Gathara, Sebastian Fuller, Mike English, Fredrick Were, Sassy Molyneux, Michuki Maina, HIGH-Q Stakeholder Representative Group, George Okello, Nelson Muriu, Stephen Kaniaru, Sharon Mweni, Anthony Murage, Daniel Mugendi, Pauline Kamau, Paul Nyamwea

**Affiliations:** 1 Health Systems and Research Ethics, KEMRI-Wellcome Trust Research Programme, Nairobi, Kenya; 2 Faculty of Epidemiology and Population Health, London School of Hygiene & Tropical Medicine, London, UK; 3 Health Systems Collaborative, Nuffield Department of Medicine, University of Oxford, Oxford, England, UK; 4 Kenya Paediatric Research Consortium, Nairobi, Nairobi County, Kenya

**Keywords:** Stakeholder engagement, Newborn unit, Global health, County hospitals, Intervention research moral, strategic and pragmatic components

## Abstract

**Background:**

Engaging relevant stakeholders throughout the research cycle is increasingly recognised as critical to conducting quality health systems research. There are few descriptions and analyses of stakeholder engagement in practice for embedded health systems research, especially those that must navigate multi-level decentralised health systems. We describe and reflect on the stakeholder engagement activities of an international multi-disciplinary programme of research focused on newborn care in hospitals in Kenya.

**Methodology:**

Our experienced project stakeholder engagement group coordinated engagement activities across multiple stakeholders, ranging from those close to the intervention to those further away with differing interests in the research. We conducted a stakeholder mapping and analysis using an engagement matrix to include national and county-level policymakers, professional communities, associations and regulators, health managers, frontline healthcare workers, patients, families and patient representative groups. Our engagement group maintained a log of engagement activities and had regular programme feedback meetings. Our analysis of stakeholder engagement drew on the Programme’s documents and meeting minutes, and on a conceptual framework which distinguishes between the moral, strategic, and practical dimensions of stakeholder engagement.

**Results:**

We engaged a wide range of stakeholders based on our understanding of their needs, interests, and concerns. We drew on the International Association for Public Participation model on stakeholder engagement, encompassing ‘inform’, ‘consult’, ‘involve’, ‘collaborate’, and ‘empower’ to inform strategies of engaging stakeholders and the need to balance moral, strategic, and pragmatic components of engagements. Although we had significant prior engagement experience and relationships at the hospitals and the counties, introducing new staff into newborn units triggered complexities that required careful consultation along the bureaucracies at the counties. Despite the counties having similar hierarchical architectures, engagement processes varied and achieved different research approval and recruitment of additional workforce outcomes across counties. There were also multiple officeholder transitions over the research period occasioned by factors in our external environment, often necessitating engaging afresh.

**Conclusion:**

Even with a carefully developed stakeholder engagement plan, an experienced team, and a landscape backed by long-term relationships and embeddedness, health research stakeholder engagement can be complex and unfold in unexpected ways. Meeting the moral, strategic, and practical potential of engagement requires flexibility, adaptability, responsiveness, continuous efforts, commitment, and adequate resources.

## Plain Language Summary

Talking to relevant stakeholders throughout the research process is getting more recognised as important to conducting exceptional health systems research. Even with this recognition, there is limited practical guidance on how to approach stakeholders, especially in studies involving layered, dynamic autonomous sub-national or local health systems. This paper describes the experiences of our research project focused on newborn care in hospitals in Kenya. The study involved interviews, observations across eight public sector newborn units and an intervention to increase staffing in four of these units.

The Project assembled a team to coordinate engagement activities involving diverse groups, including national and county policymakers, healthcare professionals, hospital staff, patients, and families. The project team adopted a stakeholder mapping approach to ensure inclusion and maintained a log of activities to adapt to emerging challenges. The inquiry into engagement activities was based on project records and a framework addressing the moral, strategic, and practical aspects of engagement.

Engaging stakeholders was key but challenging, especially with the involvement of new project staff getting integrated to work alongside existing hospital staff, which required careful coordination across various levels of the health system. These processes varied between the sub-national governance structures. Engagement was repeated and sustained especially where there were frequent changes in leadership, such as those due to elections. These experiences led us to conclude that despite careful planning and experience, stakeholder engagement in health research often unfolds unpredictably and requires ongoing effort, resources, and adaptability to achieve meaningful outcomes.

## Introduction

Health policy and systems research (HPSR) is a multi-disciplinary and applied field of research aimed at understanding and strengthening the performance of health systems. ^1^ Stakeholder engagement has gained prominence in HPSR, particularly for researchers designing, implementing, and evaluating health services interventions and programmes. ^2^ Within the health research context, stakeholders are defined as individuals, institutions, and communities with a ‘direct interest’ or influence in the process and outcomes of the research. According to Deverka *et al.* (2012), stakeholder engagement is the ‘iterative process of actively soliciting the knowledge, experience, judgment and values of individuals selected to represent a broad range of direct interests in a particular issue”. ^3^ Stakeholders in health research typically include community members, health workers, health managers, policymakers, and private sector and non-governmental actors. These stakeholders may be operating at local (micro), sub-national (meso), national (macro), and sometimes international levels.

In this paper, we describe and reflect upon stakeholder engagement for a multi-disciplinary collaborative HPSR study, the ‘Learning to Harness Innovation in Global Health for Quality Care (HIGH-Q)’ programme. The HIGH-Q programme aims to understand how technological and human resource interventions can be designed and implemented successfully to enhance the quality of inpatient and post-discharge neonatal care. The Programme is being implemented in the layered public health system in Kenya. (Additional details are provided under the study setting in the next section). Specifically, in this paper, we provide an overview of the project stakeholder engagement strategy, which is pivotal to the study. We describe the varied engagement activities and outcomes from devolved units with similar structures and mandates, as well as discuss the implications for policy and practice for similar studies in the future.

Stakeholder engagement is often seen as separate and distinct from, but informing, interactions with research participants themselves. ^4^ However, a growing set of research approaches has integrated stakeholder engagement into the study design itself, with key stakeholders and representatives of stakeholder groups and institutions targeted as key participants. ^5^ Depending on the research design and context, the engagement of relevant stakeholders can inform the research throughout the research cycle, from developing the initial research plans and questions to more detailed proposal development and implementation of the work, to completion of the research and dissemination of final research findings. In collaborative, co-designed studies or studies embedded in health systems being examined, stakeholder engagement is woven throughout. ^6^ In these ways, stakeholder engagement is integral to the anticipated pathways to impact. ^7^ Failure to appropriately engage relevant stakeholders potentially undermines the quality of learning, and interest in the study findings, and – given that stakeholders include those expected to hear about and act upon study findings - ultimately the impact of the research on policy and practice. ^4^ ^,^ ^8^

Despite the emphasis on stakeholder engagement in HPSR literature, there are few descriptions and analyses of engagement in practice for different study designs. ^9^ Available literature suggests that stakeholder engagement is often skewed towards setting up studies ^10^ and sharing study findings ^11^ with little attention to some of the complexities and tensions in managing and responding to stakeholders’ inputs throughout the research process, and (where relevant) to study teams’ withdrawal from facilities. Additionally, there is a dearth of ‘thick’ descriptions of stakeholders’ engagement across layered institutional arrangements, such as from the hospital level to higher levels of bureaucratic structures under devolved governments.

Past work by ourselves and our colleagues has highlighted the importance of purposefully selecting stakeholders to fit project needs, and of clearly defining the roles and expectations of researchers and other stakeholders from the onset. We have emphasised the importance of involving ‘across-system’ actors, including those often overlooked in such engagement activities (such as frontline health workers), and recognising and responding to the dynamic nature of stakeholder involvement over a project’s lifetime. ^1^ ^,^ ^12^ ^–^ ^16^ Engagement activities have combined those that are more and less interactive, across the continuum of engagement from consultation to collaboration. The International Association for Public Participation distinguishes between ‘inform’, ‘consult’, ‘involve’, ‘collaborate’, and ‘empower’ ^17^ with increasing depth across these forms of engagement. This categorisation has aided in planning, implementing, and evaluating stakeholder engagement and communication.

## Study setting

### The HIGH-Q Programme 2020–2025

The HIGH-Q Programme, implemented in Kenya, is a partnership between the Kenya Medical Research Institute (KEMRI)-Wellcome Trust Research Programme, the University of Oxford, the Kenya Paediatric Research Consortium, the London School of Hygiene & Tropical Medicine and the County Departments of Health. Running from October 2020 to September 2025, the HIGH-Q Programme of work comprises a co-designed, multi-disciplinary workforce intervention and evaluation aimed at improving the quality of care in select neonatal units in Kenya. The workforce intervention was built around and concurrent with a multi-country technology intervention programme known as Newborn Essential Solutions and Technologies (NEST360). ^18^ The NEST360 technology intervention is implemented in a large subset of a network of county hospitals known as the Clinical Information Network (CIN) (See Box 1 for a summary of NEST360 and CIN); the HIGH-Q programme involved a smaller subset of these NEST360 sites.

Box 1: **Programmes linked to HIGH-Q.**

**NEST360** comprises a global coalition of clinical, biomedical, and public health specialists hailing from 22 prominent institutions and organisations. The alliance’s main objective is to assist African governments in implementing a comprehensive care package encompassing cost-effective technologies, training programmes for both clinicians and biomedical technicians, and locally managed data systems, all aimed at ensuring the delivery of high-quality care for small and sick newborns. ^18^

**The CIN** is housed at the Health Services unit of the KEMRI-Wellcome Trust. Operating since 2013, the CIN is a collaboration involving the national Ministry of Health and the departments of health at the county governments. The Network has expanded to include 24 hospitals, as well as policymakers and researchers, and aims to develop and adopt evidence-based clinical guidelines for paediatric care, enhance research feedback to hospitals and serve as a vehicle for learning health systems focused on paediatric and neonatal care. ^12^ ^,^ ^16^

The HIGH-Q Programme has a specific focus on neonatal care in Kenyan hospitals. The Programme is assessing how introducing additional workforce alongside the NEST360 technologies in Kenyan hospitals affects technology adoption and quality of care in light of known and substantial workforce deficits. ^14^ The Programme’s rationale is based on the assumption that adding technologies by themselves will improve quality and safety and that the available staff can successfully implement new technologies. However, this might not be the case; hence, the research focuses on an improvement in staffing levels to assess more successful use of technologies to improve care. Completed in July 2023, the specific workforce intervention aimed to increase the number of nursing and ward assistants in newborn units (NBUs). ^19^ Specifically, the intervention involved the employment of three additional nurses and three ward assistants in each of the four-county hospital NBUs for 15 and 7 months, respectively. Sick newborns in NBUs require continuous monitoring and treatment, but low nurse-to-patient ratios compromise care and have been linked to an increase in mortality. While adding more skilled nurses could improve care quality by reducing missed tasks and strengthening teamwork, it remains a challenge due to underfunded health systems. Nurses also spend time on lower-skilled duties, such as cleaning equipment, changing linens and diapers, and assisting mothers with breastfeeding, tasks that could be delegated to ward assistants.

Salaries for the additional intervention staff were paid by the HIGH-Q Programme for the research period, but the staff were employed and line-managed by the hospitals. The other HIGH-Q objectives examine health workers and mothers’ experiences of newborn care, how to better support the delivery and integration of post-discharge care for families, the governance process of introducing technologies and service delivery innovations and infection prevention and control in these NBUs. Primary data collection includes structured and unstructured observations of care and technology use at NBUs and interviews with healthcare professionals (at all levels of the health system) and with the carers of sick babies. Table 1 summarises HIGH-Q Programme work packages, research questions and methods. The Workforce intervention was assessed under work packages 1a and 1b.

**Table 1.** HIGH-Q Programme Work packages, research questions, and methods.

WP1a: Health workforce intervention and indicators of quality of newborn care Question: If staff-to-patient ratios are increased by adding new staff in NBUs, does this improve quality of care?		
Methods	Sites	Sample (accomplished)
Direct observation of care using the nursing care index tool, tracking workforce changes using staff Rota	4 intervention county hospitals 4 Non-intervention County hospitals	Babies observed = **1,302** (948 at intervention and 354 at non-intervention sites), **10,379** hours of observation in intervention sites (+3,938 hours at Non-intervention sites)

WP1b: A process of delivering and implementing technologies and uptake factors Question: When new technologies are introduced in NBUs, what effects does this have on staff and families?		
Methods	Sites	Sample (accomplished)
Observation, informal and formal interviews with health workers and mothers, as well as document review	4 intervention county hospitals	Mothers interview **n = 38,** Nurses Interviews **n = 101** House of observation **n = 3500**

2A: The process of post-discharge neonatal care and how innovations can improve pathways to care Question: *How can we work with hospitals, staff, and families to co-design better post-discharge care services risk for newborn and their families?*		
Methods	Site	Sample (accomplished)
Observation, informal and formal interviews with health workers and mothers as well as document review	4 intervention county hospitals	4 co-design workshops, 15 interviews with healthcare providers, 21 interviews with mothers. Observation (160 hours in hospitals, 34 hours in homes)

Wp3: The governance process of introducing technologies and service delivery innovations Question: *How do global, national, and local governance structures and processes interact to shape the introduction and oversight of new and existing medical devices in Kenyan hospitals?*		
Methods	Site	Sample (accomplished)
Informal and formal interviews with health workers, health regulatory, and NEST 360 managers and document review.	4 intervention county hospitals, 1 non-Intervention county Hospital, 2 additional hospitals.	32 interviews with health workers and NEST 360 staff. 3 FGDs with health workers, 3 interviews with national managers, and informal discussions with national and county stakeholders.

In Kenya, health care is devolved from the national government to 47 semi-autonomous sub-national governments (commonly known as counties). ^20^ Under this governance arrangement, county governments have an executive, administrative, and legislative mandate over their areas of jurisdiction. From the early study planning stages for HIGH-Q, it was recognised that stakeholder engagement was needed at national, county, hospital and newborn unit levels, and should build upon and consider wider CIN and NEST360 activities, as well as any other research that might be ongoing at these sites during this period.

## Methods

An experienced programme stakeholder engagement group was established at the onset of HIGH-Q to coordinate stakeholder engagement. The stakeholder engagement group constitute the Programme’s principal investigators and researchers, the majority of whom are from or have lived and worked in the Kenyan health system for over a decade. The KEMRI-Wellcome Trust-based research group is coordinated by a project manager, the first author (KK), who himself has health system management experience and master-level training in public administration.

Stakeholder engagement activities were integrated at the various stages of the Programme, from proposal development to the pre-intervention period and during the various phases of the Programme’s implementation.

**Figure 1.**
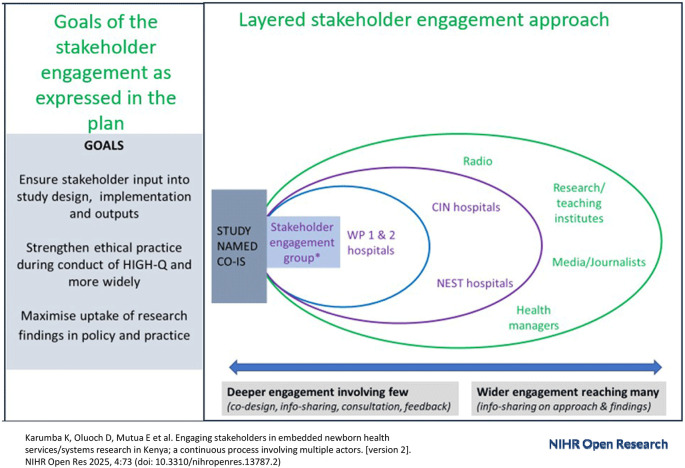
The HIGH-Q Community and Stakeholder engagement framework goals and the components of engagement.

An initial engagement strategy consisting of goals for engaging stakeholders was developed at the proposal development stage. This strategy adopted a Rainbow diagram for stakeholder identification, adapted from Chevalier and Buckles (2008). ^21^ Under this model, stakeholders were identified and placed in concentric layers depending on influence, interest and how close they were to the NEST360, and the HIGH-Q research as presented in Figure 1 below. Identified stakeholders were categorised and layered from those closer to the research to those further away. This adjacency to research was informed by direct and frequent interactions with the technological and workforce intervention and the HIGH-Q data collection. For example, mothers and frontline nurses operating within the NBUs were placed in the inner layer, and those far away, such as national policymakers, teaching institutions, and the media, were placed on the outer layer. This process yielded multiple stakeholders who included the national and county-level policymakers, medical and nursing professional communities, professional association regulators, health managers at various levels of the health system, frontline healthcare workers in hospitals, patients, family members, and patient representative groups. These stakeholders, especially those close to the research, were engaged throughout the HIGH-Q research process. After the mapping exercise, an engagement matrix analysis was conducted to understand who the stakeholders in the planned research are, their composition, their interests and concerns, and how they are likely to influence the study’s success. In Appendix 2, we provide a template that was developed in-house and adopted for the engagement matrix analysis. This matrix is a balance between the stakeholder attributes and the goals of the Programme. The appendix also provides a summary explanation of the concepts adopted to analyse stakeholders- stakeholder group category, composition, closeness to the study, interest of stakeholders, programme interest, and objectives of engagements. The matrix informed the implementation of key stakeholders’ activities, such as the articulation of the engagement activities, key messages, tools of engagement and timelines. Stakeholders were reached through official letters signed by the principal investigators and posted via courier. Follow-ups of the official correspondence were done through emails and telephone calls with contacts retrieved from a CIN contacts list shared across projects in the network working with the same CIN site hospitals. Key messages were crafted based on specific aims of stakeholder engagement encompassing ‘inform’, ‘consult’, ‘involve’, ‘collaborate’, and ‘empower’ as advanced by the International Association for Public Participation. ^17^

Stakeholder engagement meetings were held in person at various national and county offices, in conference meeting rooms and in hospitals, complemented by follow-up online meetings, telephone calls, email correspondences, posted mails and through a newsletter. Feedback on stakeholders’ priorities, concerns and on complex scenarios emerging from the engagements was relayed to the principal investigators and the wider scientific team through regular meetings, and where applicable, to individual team members, the NEST360 and the CIN.

The entire engagement process was carefully documented and tracked in a log/diary by the project manager. The log consisted of a spreadsheet where entries were made for the date when an engagement activity took place, description of stakeholder engagement activity, the mode of engagement, the category of targeted stakeholder, the county targeted, the stakeholders’ messages and the frequency of engagement. A template of the log is provided in Appendix 3. To write this paper, we drew on recorded activities and meeting minutes gathered between October 2021 and February 2024, a period covering the Programme’s proposal development stage, study introduction, fieldwork, initial research feedback to hospitals and the study withdrawal.

### Synthesis and Interpretation of Learnings

To support our reflections on the impact of the Programme’s stakeholder engagement activities, we have applied ideas from Kujala, Sachs, Leinonen, Heikkinen & Laude (2022)’s conceptual framework, which proposes engagement activities to consist of moral, strategic, and pragmatic components. ^22^ Their framework is a response to what is observed by the paper as a fragmented approach to analysing stakeholder engagement, which hampers research progress. The **moral component** involves considering the ethical dimensions of how the operations of an organisation affect individuals and groups rather than focusing on organisational interests. In the context of our research, we consider, for example, how to engage particularly marginalised stakeholders, how to ensure respectful interactions with all stakeholders, and how to ensure the research is made meaningful to diverse stakeholders. The **strategic component** is concerned with how managers distribute scarce resources among stakeholders in ways that guarantee value creation for the organisation and the achievement of its objectives. In the context of our research, for example, we recognise that we have deliverables to ourselves and our funders with regard to the quality of research and our contribution to knowledge. The **pragmatic component** could be seen as working across the moral and strategic components. Drawing on Chinyio and Akintoye (2008), ^23^ and Forsythe, Ellis, Edmundson *et al.* (2015), ^9^ we considered these as pragmatic activities to build and sustain connections, including employing negotiations, making trade-offs and adjusting to the practical requirements of stakeholders.

Under this approach to reflection, stakeholder engagement activities in the Programme were assessed based on meeting the three goals of engagement: the moral, the strategic and the pragmatic components by analysing the elements of the of Programme specific activities against the three components.

## Results

We begin by describing our initial strategic plan for the stakeholders’ engagement, followed by sharing some of our experiences in navigating interactions across a diverse and evolving stakeholders’ engagement landscape. We also outline the outcomes of our engagement across counties. Finally, we outline our approach to the dissemination of research findings and exit strategies after project completion.

### Developing an initial strategic plan to guide stakeholders’ engagement

During the proposal development and the programme’s setup phases, the investigators formulated an initial framework for engaging stakeholders. This framework encompasses engagement objectives, detailed stakeholder mapping, and the nature and content of engagement activities ( Figure 1). The overall interrelated goals of stakeholder engagement were to contribute to 1) good quality science through inputs into study design, implementation and outputs; 2) ensuring ethical practice throughout the research process; and 3) maximising the uptake of research findings in policy and practice.

The engagement framework covered a full spectrum of activities. At one end of the spectrum were deep engagements involving fewer people in more interactive ways. Activities include engaging stakeholders in consultative meetings to co-design elements of the research and get advice on the stakeholders’ engagement plan itself, as well as on how to conduct the research. At the other end of the spectrum, there are wider engagement activities aimed at reaching many people through inevitably less interactive activities. Examples include radio programmes, video outputs, newspaper articles, and policy briefs.

At the heart of the engagement activities, and to advise on all our undertakings, we set up an international and a national advisory expert group with diverse expertise. The international expert group includes seven stakeholders with global health research experience and content expertise in medical sociology, health economics, organisational analysis, health workforce and systems, infectious diseases epidemiology, regional nursing policy and advocacy. The national expert group consists of eight individuals with representation from the national Ministry of Health, medical schools, medical and nursing professional communities, professional association regulators, and patient representative groups. These groups were consulted together at least annually, supplemented by periodic interactions with members as needed. The expert group contributed towards the study design. During the early stages of the study, the research team received valuable input from the members of the expert group that helped with research approaches and execution. An example of key advice on the sequence of additional nurses and ward assistants’ intervention is highlighted below:

*“I would rather add the nurses first and then add the assistants later. The nurses will be supervisors to the assistants, so it is better that they are first orientated to enable them to be more useful. So that we can say the changes are due to the registered nurses”*

(N EG 1)

### Implementing stakeholder engagement activities across a complex and expanding range of stakeholders

**Figure 2.**
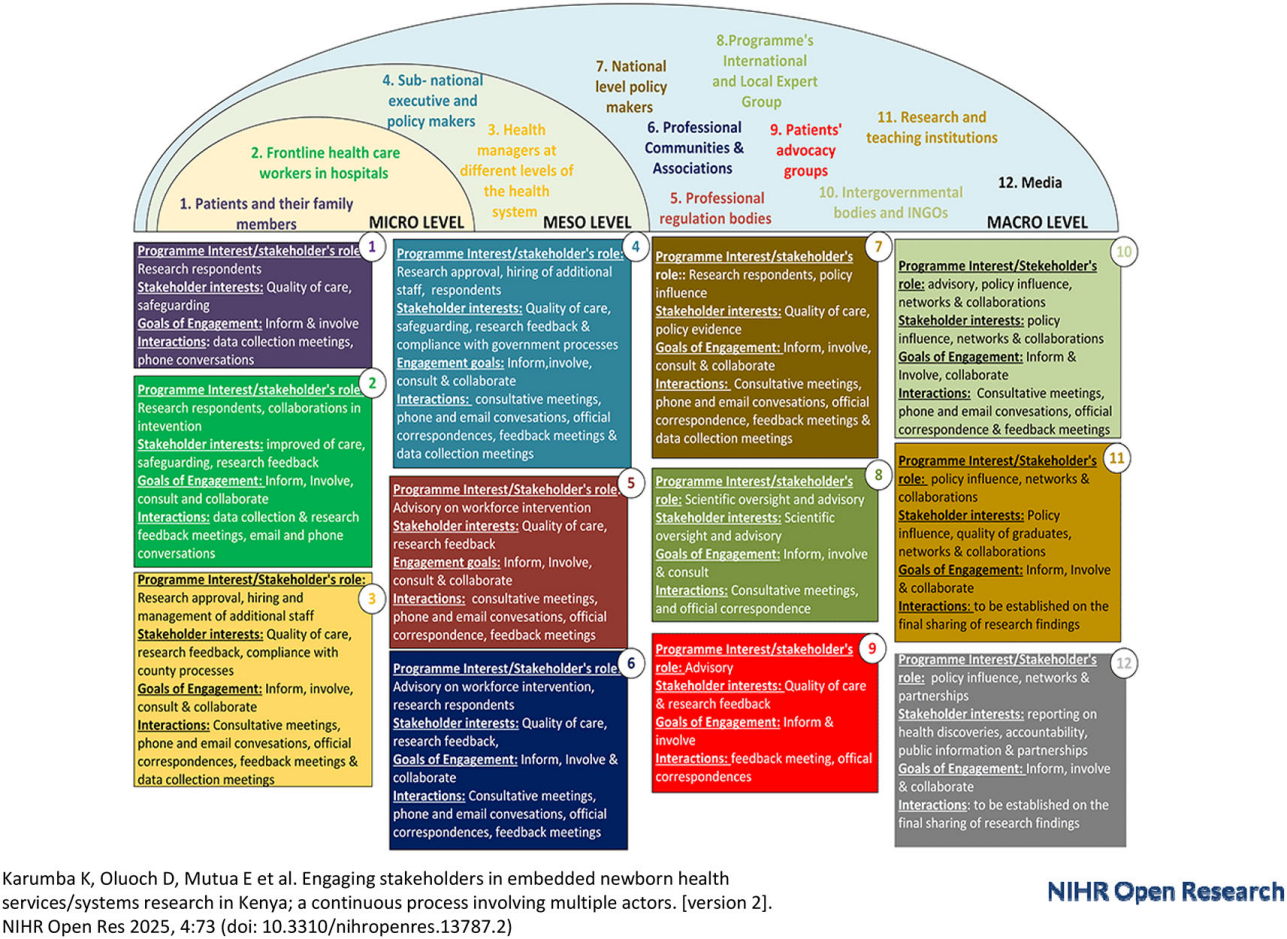
Presentation of multiple and layered stakeholders engaged, showing differing interests.

The process of identifying stakeholders using the engagement matrix yielded multiple stakeholders who included the national and county level policymakers, medical and nursing professional communities, professional association regulators, health managers at various levels of the health system, frontline healthcare workers in hospitals, patients, family members and patients’ representative groups. These stakeholders, especially those close to the research, were engaged throughout the HIGH-Q research process based on our understanding of their needs, interests, and concerns ( Figure 2). Drawing on the International Association for Public Participation distinctions described above, we ***informed*** all stakeholders by providing study details and our plans to help them comprehend the research issues and support further engagement activities. We also ***involved*** representatives of all stakeholders (including research participants) to ensure their concerns were understood and considered in research activities and study learning. We ***consulted*** with representatives of many stakeholder groups to gather input regarding our plans, including through our advisory groups, and had formal ***collaborations*** with stakeholder groups drawn from the counties and hospitals. We sought to ensure that the HIGH-Q research was as responsive as possible to stakeholders’ needs and priorities, and we would not have been able to proceed if gatekeepers such as regulators and county and hospital managers had not approved the study. Nevertheless, the final decision-making on the study details remained with the study team. In these ways, we were continuously balancing ethical, strategic, and pragmatic elements of stakeholder engagement. At the workforce intervention level, the recruitment approach followed county procedures as described in a subsequent section. At the NBU level, nurse managers were fully empowered to manage duties and performance of additional nursing staff.

**Figure 3.**
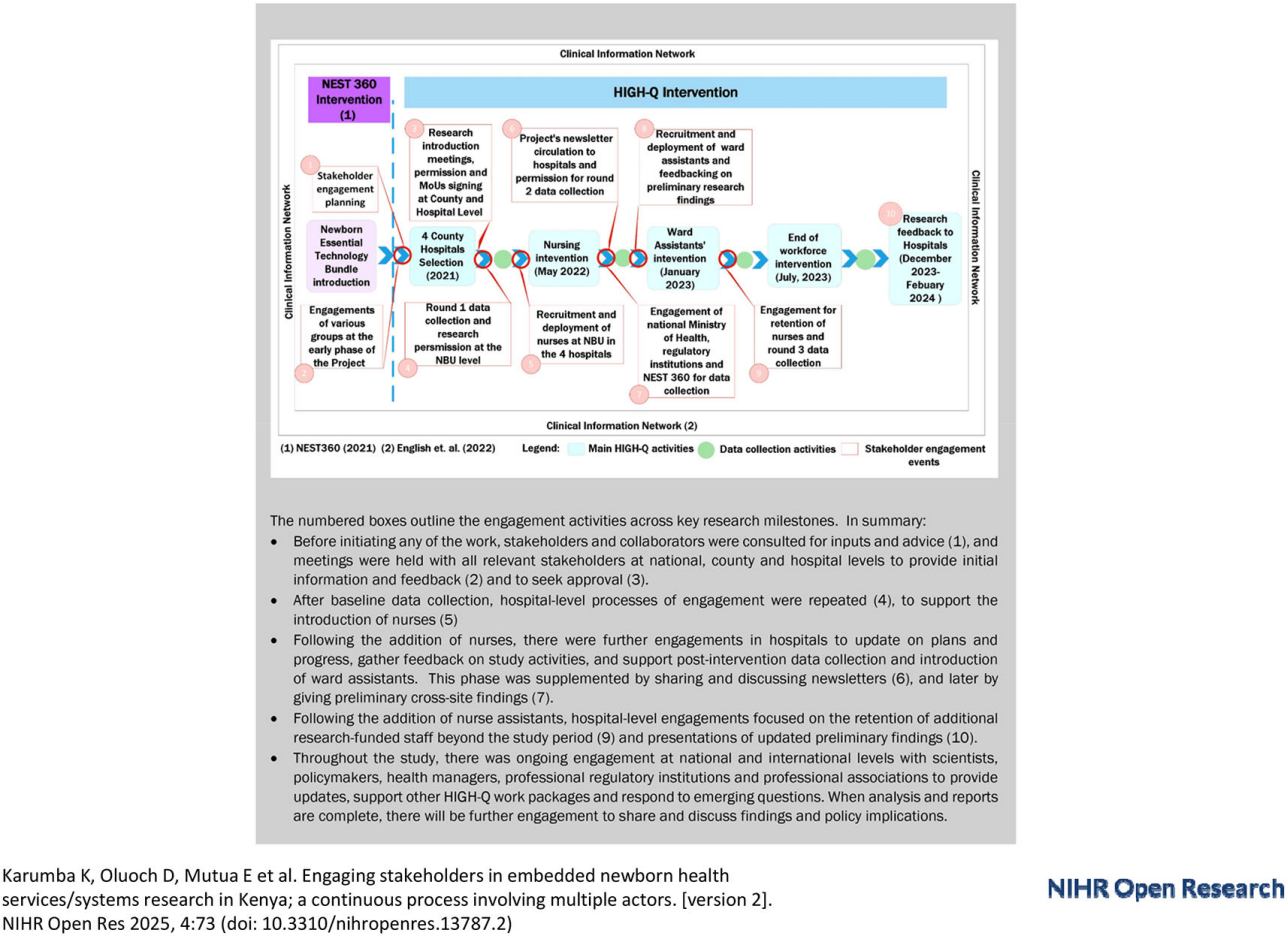
A summary of the HIGH-Q Stakeholder engagement against Programme’s milestones.

**Figure 4.**
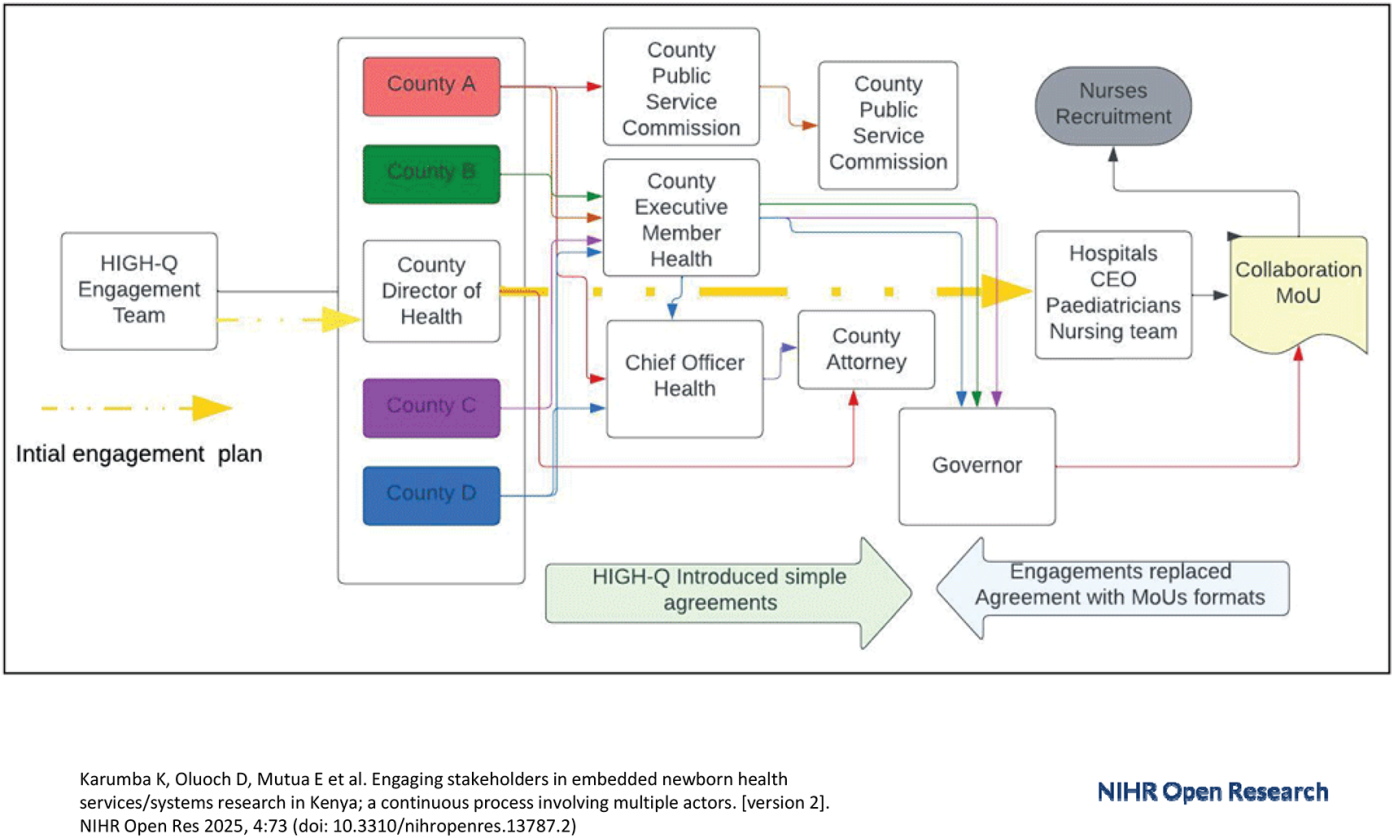
A presentation of the County and Hospital structures and the evolving engagements experienced by the HIGH-Q Programme.

As presented in Figure 3, there were three main observations from this work. First, the bulk and the backbone of stakeholder engagement focused on four intervention hospitals. We were aware that engaging the leadership and health workers is critically important. Our initial engagement plan targeted the county directors of health (DoHs) as the first point of entry, followed by engagements at the county hospitals and down the hierarchy to the NBUs level. However, the workforce component significantly expanded the complexity of our engagement processes, as outlined in Figure 4.

Our second observation is that we had to respond to field questions and realities with many informal or unplanned follow-up engagements or activities in response to issues raised by the study teams or stakeholders. Examples include:

• Ward staff raised informally that there were too many research staff conducting observations or interviews in a facility at one time, potentially impacting data collection and patient care (co-authors EM, DO, SF, MM, SM) are assessing these concerns and other observed ethical issues arising from conducting the HIGH-Q research. In response, HIGH-Q fieldwork was reorganised, and new ways of working were shared with hospital stakeholders.
• Hospital managers mentioned that overlaps and links between NEST360, CIN, and HIGH-Q were unclear, potentially leading to confusion. In response, communication was reorganised, rewritten and coordinated to clarify the distinctness, the differences and the overlaps of the three Programmes. Specific stakeholder feedback was channelled to the relevant Programme.

We observed that while some of these issues could be considered primarily practical, strategic, or moral, there were often interactions and interplays between the components. For example, having too many research staff at a ward had practical implications (difficult to collect data) and moral implications (disruption to routine ward services offered by overwhelmed staff caring for vulnerable children). Our third observation was that some stakeholder groups were more challenging to engage in terms of reaching them and achieving sustained involvement than others. For instance, our engagement with parents was primarily through interviews and observations, preceded by consent and informal interactions. Linked to research governance, our study was subject to review and approval by the National Commission for Science, Technology, and Innovation (NACOSTI), and our international researchers were required to apply for and obtain research permits from the Immigration Department. Still, on research governance, we had more interactions with nursing regulators/professional bodies such as the Nursing Council of Kenya (NCK) and the National Nursing Association of Kenya (NNAK). Our engagement with these regulatory institutions involved following all requirements at international, national, and local levels for initial approval and, where necessary, annual reports and renewals are required. At the data collection level, we reached out to a range of regulators of health research and technology to conduct interviews, with varying success.

As part of connecting to an even wider audience, we plan to engage with teaching institutions and the media as part of the next phase of stakeholders’ engagement.

### Different approaches and outcomes between counties with similar architecture.

In the county governance establishment, there were several important officeholders to consider in any hospital-based engagements (See Box 2 on County Governments and the Departments of Health structure relevant to the study, adapted from the County Government Act, 2012 ^24^ and the Transition to Devolved Government Act, 2012 ^25^). In the HIGH-Q Programme, the workforce intervention triggered a more complex set of engagements than is typically required for more observational or descriptive research. Figure 4 illustrates our original linear simple partnership plans in the centre, represented by the broken yellow line arrow. At the engagement stage, this approach morphed into many referral contacts, with many people to talk to and get permission from and eventually led to the conversion of simple agreements into complex memoranda of understanding (MoU). To get permission and approvals, we engaged with the chief officers, the county executive committee member, the county public service board, the county legal offices, the county secretary, and the governors. These engagements differed across counties, leading to differences in the way intervention staff were recruited, despite the similarity in county hierarchical and administrative architecture.

Box 2: **County Government and the departments of health structure relevant to the study.**

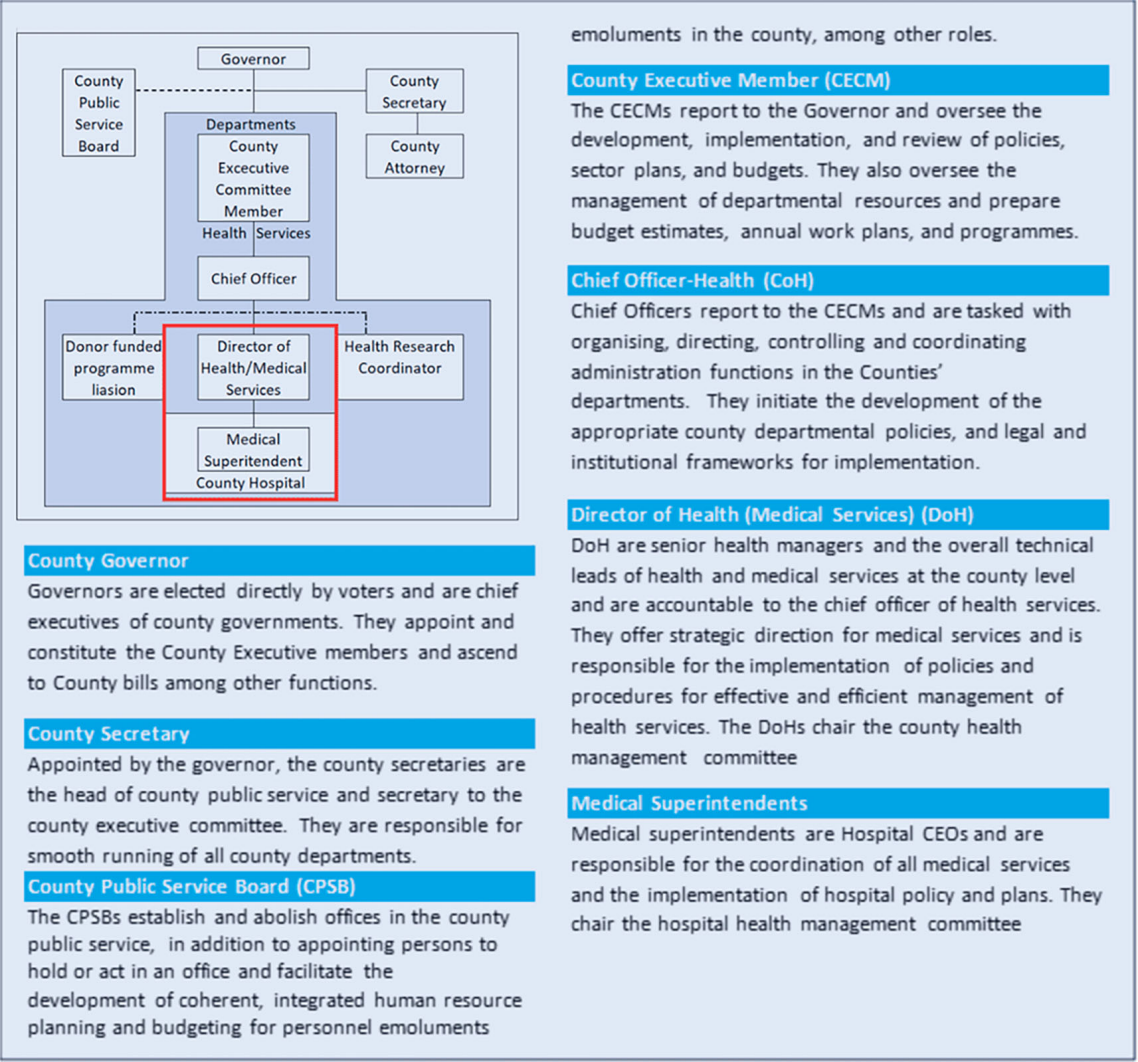

Other notable differences were in the frequency and duration of research approvals and for the other research-related milestones in the four counties. Table 2 below presents quantitative data on the frequency and the duration of engagements with different county officials in the pre-intervention (PRE-I) period, comprising 7 months, and during the intervention (DUR- I), comprising 15 months. The pre-intervention period was before the MoUs signing and the recruitment and deployment of additional staff. The intervention period consisted of deploying staff working in the units, and the data collection period. The number of engagements in this period with county and hospital-level senior office holders varied across the counties. The combination of methods employed for engagement consisted of face-to-face, phone calls, email correspondence, and letters. A majority of county and hospital executives were engaged directly and at varying durations (presented numerically in brackets and consisting of weeks of engagement from initial contact to sharing of findings) and frequencies (also presented numerically). Those closer to the intervention (the medical superintendents) were engaged multiple times, while those further from the intervention (the governors and the CECMs) engaged less often.

**Table 2.** A presentation of the frequency and Duration of stakeholders’ engagement activities, before and during the intervention.

County and Hospital personnel engaged	Frequency of engagement activities and duration of engagement in weeks in brackets							
	County A		County B		County C		County D	
	PRE-I	DUR- I	PRE -I	DUR- I	PRE-I	DUR -I	PRE-I	DUR-I
Governor	N/A	N/A	1 (6) *	N/A	1(3)	N/A	1 (2)	N/A
County Secretary	1 (5)	N/A	N/A	N/A	N/A	N/A	N/A	N/A
County Public Service Board	14(30)	12(8)	N/A	N/A	N/A	N/A	N/A	N/A
County Executive Committee Member Health	1(1)	2 (3)	1(6)	N/A	3(10)	2(3)	(3)5	2(3)
County Legal Officer	N/A	N/A	1(1) *	N/A	3 (3) *	N/A	N/A	N/A
Chief Officer Health	4(3)	1(1)	N/A	N/A	N/A	N/A	N/A	N/A
County Director of Medical Services	22(12)	N/A	46 (30)	7(65)	12(30)	(17)65	N/A	N/A
Medical Superintendent	4(30)	5(65)	16(30)	23(65)	7 (30)	8(65)	17(30)	11(65)

* Engaged indirectly.

The differences in engagement pathways between counties were also reflected in the execution of MoUs and recruitment of staff ( Table 3). The pathways differences were because of proactive incorporation of county stakeholders’ input into the engagement process and consultations on how best to implement the study as collaborators. For example, as presented in Figure 4, the Programme agreement proposals were expanded and converted to MoUs and included additional clauses and signatories. We assessed MoUs attributes according to the number of signatories, the number of articles, and the duration in weeks from their initiation to full execution. We see that County A added an extra clause on recruitment by the County Public Service Board (CPSB), and County B was directly coordinated by the county director of health (DoH), who worked closely with the County’s legal team to develop and sign a complex MoU with multiple clauses. The process at County B took double the time of Counties A and C. The extra layer of engagement process involving the CPSB was an uncharted route before and presented tension and uncertainty with budgetary implications for the advertisement of nurses’ jobs in a newspaper with national circulation. However, these concerns were well mitigated with an efficient internal consultative process, strategy, and open engagement with the Board. Still under recruitment, County B deployed intervention staff in other departments and utilised its own staff for the intervention. Though this was a noble initiative for sustainability, the Programme had to carefully relay this feedback to the newly recruited staff whose contract indicated they would work in NBUs and would be trained before deployment.

**Table 3.** A presentation of the dynamics of MoUs execution experiences in the four counties.

	County	County A	County B	County C	County D
** MoU Attributes**	**Highest ranking signatory**	County Secretary	Governor	CECM	COH
	**No. of County Signatories**	5	2	3	2
	**No. Articles in MoUs**	8	23	7	7
	**Duration to full execution (weeks)**	7	14	7	9
**Recruitment of staff**	**Approaches to recruitment of Nurses**	Through CPSB	Through the Programme with the Director of Health represented in the interview *	Through the Programme with the Director of Health represented in the interview	Through the Programme with the medical superintendent represented in the interview
	**Approaches to recruitment of Ward assistants**	Through CPSB	Through the Programme with candidates getting nominated by the hospital	Through the Programme with candidates getting nominated by the hospital	Through the Programme with candidates getting nominated by the hospital

* County B deployed recruited staff in other departments and utilised existing staff for the intervention.

Our experiences underscored the importance of being able to be flexible and responsive in our engagements and in the research plans. Notably, the entire process above was also complicated by routine job (re) postings and by national general elections. At the governor level, three of four governors and all four executive committee members (CECMs) at the Department of Health in the four counties vacated office after the national general elections, and in one of the counties, there were 3 different CECMs during the engagement period. All four chief officers at the departments of health (COHs) left after the elections. During the engagement period, all hospitals registered a transition in the office of the medical superintendents. The first transition of medical superintendents happened only one month after the commencement of engagement at County C, and the last a month before the end of the engagement period. These transitions of various officeholders necessitated renewed engagements, especially where the new officeholders were closely involved in the implementation, such as medical superintendents. In Counties A&B, the transition for the position of medical superintendent happened even before the MOUs had been signed.

### Engagement for research feedback and study withdrawal

As illustrated in Figure 3, we undertook two cycles of research feedback at the four county hospitals during the engagement period, with further feedback and discussion of findings planned over the next 6–12 months.

The first research feedback meeting took place between November and December 2022, six months into the nursing workforce intervention and just before the introduction of ward assistants. The objective of this feedback was to give progress updates and preliminary research findings from HIGH-Q’s baseline survey research. The findings were anonymised across all four sites. The target audience included the medical superintendents, paediatricians, nursing leaders, and various departmental staff. The feedback sessions provided opportunities for hospitals to understand the HIGH-Q’S qualitative work and the consent process, and to promote discussion on areas of care the hospitals were eager to improve on. Stakeholders were keen to have hospital-specific data presentations in the future as a reaction to data anonymisation approach adopted for this initial round of feedback.

The second set of feedback occurred between December 2023 and February 2024, a year later, after completion of all the fieldwork, and following further data analysis. Before this round of research feedback, the research team spent considerable time carefully preparing for the feedback and crafting appropriate messages to hospitals. Hospital-specific data was provided together with comparative anonymised data from other hospitals. Sensitive content was provided feedback in careful ways. The NBU hospital teams were interested in discussing the clinical data, but were also concerned about the withdrawal of intervention staff once the study was over.

In response to the NBU team’s request to support with advocacy for the retention of intervention staff, and as part of the withdrawal plan from hospitals, we conducted an additional extensive set of engagements with county stakeholders at the departments of health in all four counties. We corresponded with all county and hospital executives previously involved in the study approval process - county secretary (County A), CECMS (all counties) CoH (Counties A&C) DoHs (all counties), and medical superintendents (all counties). Our engagements involved a brief highlight of existing partnership and contractual arrangements for the HIGH-Q programme, an update on the status of workforce enhancement and the short-term employment of intervention staff, an assessment of the contribution of the additional workforce to the quality of newborn care at the NBUs, and finally, exploring opportunities to retain and absorb the project nurses. These efforts culminated in a brief face-to-face meeting with three of the four CECMs at a CECM forum. Our interest in having continuity of the nursing workforce conflicted with the position of CECMs. The feedback from the CECMs cited county human resources practices which promote a competitive recruitment process, leaving no room for the direct absorption of staff from the Programme into the county public service. Options for inviting the HIGH-Q Programme nurses to be part of recruitment once public funding for staffing at the county was discussed, with promises from the CECMS that staff would be offered opportunities through an open recruitment process. These discussions, however, took place at a time of a major nationwide problem with recruiting new health staff linked to national budget caps with direct implications for the County governments. The Public Finance Management (National Government) Regulations, 2015 (Kenya) ^26^ imposes a limit of 35 per cent expenditure on county staff salaries and benefits against total county revenues. According to the latest report available, on average, the County governments have exceeded this limit. ^27^

## Discussion

Stakeholder engagement in HPSR has shifted beyond access and compliance requirements to embrace ethical principles such as ensuring respectful interactions, seeking to maximise benefits and minimise inconvenience to all involved in research studies. Involving key stakeholders at every stage of the research process is now widely acknowledged as essential for producing high-quality, impactful health policy and systems research. For the HIGH-Q Programme, the study design, past experiences and evolving nature of engagements contributed to extensive engagements with multiple international and local stakeholders.

In engaging multiple stakeholders, we found the International Association for Public Participation’s distinctions between ‘inform’, ‘consult’, ‘involve’, ‘collaborative’, and ‘empower’ valuable. Potthoff *et al.* (2023) observed that the IAPP framework classification aids in the quest for conceptual clarity. ^28^ The framework supported our engagement work, specifically in refining our objectives and in designing communications for specific stakeholders, helping us achieve an impactful engagement process and study approvals.

Each of the stakeholder engagement goals ( Figure 1), and many of the engagement activities ( Figure 2) and ( Figure 3) have moral, strategic and pragmatic elements. For example, the engagement of international and national experts to ensure good quality research is planned and conducted, and in helping guarantee that the research is context-relevant, potentially impactful and conducted in ways that are sensitive to hospitals, their environments and cultures could be viewed as strategic. Similarly, the engagement of counties and hospitals’ senior staff for initial approvals and later to give study feedback and advocate for the retention of intervention staff had elements of all three components. The potential value of distinguishing between these elements in a context like ours could be in the research team regularly reflecting on the balance across the three components, and whether this remains appropriate across the shifting lifespan of a programme.

Prebanic and Vukomanovic (2023) observed that the engagement of project stakeholders can be highly complex because of stakeholders’ conflicting interests, which can lead to time and cost overruns. ^29^ In our experience, complexity arose less from conflicting interests than from the need to seek buy-in and inputs from a large and expanding set of stakeholders at different levels of the health system hierarchy, and to having to continuously re-engage with stakeholders as the study progressed. This complexity in engagement was evident even with a well-established, long-standing relationship like the CIN network and freshly instituted relationships like those of the HIGH-Q programme. The complex pathway route was found to be necessary in the quest to maximise stakeholder input and interest and aimed to strengthen the potential for uptake of study learning into policy and practice in ways that sought to balance the strategic, moral, and practical elements of engagements as our work and the contexts evolved and shifted.

Our engagement’s frequency intensified, and duration stretched due to the workforce intervention component. The added project staff were required to operate as part of normal hospital duties, with output contributing directly to hospital service delivery. In Kenya, a trend of ‘recentralization within decentralisation’ has been described by Barasa *et al.* (2017), whereby hospitals have lost some of their pre-devolution autonomy to county-level departments of health, higher up the health system hierarchy. ^30^ For the human resource management function, part of hospital autonomy is surrendered to the county public service boards during the process of institutionalising devolution. For these reasons, it was essential to engage county Departments of Health and, in specific instances, the county public service board, the county secretary, and the governor before intervention staff were employed. Strict adherence to county human resources guidelines and prescribed recruitment path has a direct impact on achieving the study objectives as well as the uptake of research findings.

The variations of engagements and pathways to engagement for counties with similar structures can be attributed to executive and legislative autonomy of individual counties, but also to the management style of various bureaucrats within the devolved structures. Various fundamental county governance styles in Kenya include but are not limited to, bureaucratic and political, ^31^ participative, ^32^ centralised and decentralised, ^30^ innovative, and private-sector-driven. ^33^ Leadership styles, local contexts, political realities and institutional capacity have shaped these styles.

Working with evolving engagements that laid bare different pathways for county engagement and county variations from the engagement process did not present a problem to the HIGH-Q engagement team and researchers. All hurdles encountered were mitigated by an understanding of the devolved governance structures by the project manager and through sustained consultations with the county executives. Murphy *et al.* (2021) have described the importance of acquiring an exhaustive contextual understanding before engaging in the implementation of a global health project. ^34^ Our findings support and extend these arguments. Our county engagement outcomes were cushioned by our long-term, embedded relationships supported by a rich contextual understanding. Importantly, we continued to learn about the nuances of context-specific engagements, especially involving county governance structures that were looped into engagement plans. Additionally, we emphasise the value of a well-resourced, continuous, and responsive stakeholder engagement process. Central to responsiveness was the study team’s willingness and ability to respond to stakeholders’ formal and informal requests for information.

Adhikari *et al.* (2019) have highlighted the importance of working through local hierarchies to seek permission for research. ^35^ Planners of multi-site programmes like HIGH-Q may adopt a singular strategy in the process of stakeholder engagement, where such hierarchies and structures are similar on paper. Our findings on the variation of time taken, the structure of the MoUs, the number of individuals engaged and the route of the engagements in the devolved governance hierarchy demonstrated that this may not be the case and call for stakeholder engagement planners to consider the potential of such variations and their implications for project timelines. Our work with governance hierarchies was also occasioned by the transition of several office holders. Such transitions involving high turnover of public sector bureaucrats make established relationships, and thus stakeholder buy-in transient. ^35^ For the HIGH-Q programme, this transition was largely due to general elections, which happened one year into the Programme’s implementation, as well as from more routine administrative postings. These local-level transitions remind practitioners and project designers of the need to pay attention to the ever-changing contextual landscape and transitions such as those involving the movement of populations, especially for longitudinal studies.

## Conclusion

Stakeholder engagement has gained prominence in HPSR among researchers and funders, with its integration going beyond project kick-off to implementation, project close-outs and knowledge translation. Engagements aimed at introducing studies with interventions targeting a key pillar of a health system, such as human resources in a highly regulated setting, can set in motion complex and unpredictable processes shaped by hierarchical structures, bureaucratic requirements, and shifting power relations. Such processes can lead to engagement hurdles that have the potential to affect project implementation timelines and may result in poor research outcomes. Navigating complex engagement landscapes can be aided by elaborate stakeholder engagement plans, executed by experienced engagement teams that incorporate lessons from frameworks and contextual experience. Also, importantly, such engagements must be approached with flexibility, responsiveness, continuous efforts, commitment, and adequate resources.

Meaningful engagements cannot only be measured by outcomes such as approvals and permission to conduct research in complex environments but must be regarded as an avenue to seek stakeholder input into study design, strengthen ethical practice and maximise research update in ways that fulfil the moral, strategic, and practical potential of engagements. In the case of the HIGH-Q Programme, two eternal factors, the COVID-19 lockdown and the national general elections, posed a risk to the intervention implementation timelines. However, the engagement strategy and the resulting support from stakeholders helped immensely to deliver the project within the grant period.

## Ethics approval

Ethical approval for the study was obtained from the KEMRI Scientific and Ethics Review Unit, Ref: KEMRI/SERU/CGMR-C/229/4203, dated 27 May 2021, with annual renewals on 13 May 2022 and 18 May 2023. Approvals were also received from the County Departments of Health of the four participating intervention hospitals.

## Consent

This report is based on the description of the stakeholder engagement process in newborn health services by the Programme’s stakeholder engagement team rather than a research study. As such, no consent was required to analyse Programme documents used in the study.

## Data availability statement

Appendices 1 & 2 data on the HIGH-Q Programme engagement matrix analysis template and the HIGH-Q Programme Stakeholder Engagement Logs sample, respectively, are openly available in Figshare at https://doi.org/10.6084/m9.figshare.32404506.v1 as extended data. ^36^ The data used for this paper is available on request. Access applications can be made through the Data Governance Committee with details available on www.kemri-wellcome.org, or by email to cgmrc@kemri-wellcome.org.
