# Supplementary material for: Engaging stakeholders in embedded newborn health services/systems research in Kenya : continuous process involving multiple actors
Source: NIHR Open Res. 2026 Jun 9;4:73. Originally published 2024 Dec 2. [Version 3] doi: 10.3310/nihropenres.13787.3 (PMC12887380; doi:10.3310/nihropenres.13787.3)
Supplement: Peer review history

# Reviewer report: Reviewer response for version 2

**DOI:** 10.3310/nihropenres.15455.r39273

**Published:** 2026-02-09

**Authors:** Uta Lehmann

**Affiliations:** 1 University of the Western Cape, Cape Town, South Africa

I see that the manuscript has already been through one round of review and revision, which has been conscientiously implemented. I therefore will not make detailed proposals regarding structure and focus. In my view it is a paper, which covers a very important and under-discussed aspect of HPSR research, and generally research in applied settings. The authors have explained processes and methods and how these evolved with great care - sometimes in so many details that, as an outsider, one is in danger to get a bit lost. I would have liked to have seen a more succinct discussion and conclusion, with more and clearer lessons for researchers in similar settings, beyond the fact that stakeholder engagement is complex and somewhat unpredictable and needs to be tailor-made and adjusted throughout the research process.

 I recommend that the paper be indexed now. However, it does need a careful copy edit, as there are a few minor grammatical errors and incomplete sentences.

Is the rationale for developing the new method (or application) clearly explained?

Yes

Is the description of the method technically sound?

Yes

Are the conclusions about the method and its performance adequately supported by the findings presented in the article?

Yes

If any results are presented, are all the source data underlying the results available to ensure full reproducibility?

Yes

Are sufficient details provided to allow replication of the method development and its use by others?

Yes

Reviewer Expertise:

HPSR, HRD

I confirm that I have read this submission and believe that I have an appropriate level of expertise to confirm that it is of an acceptable scientific standard.

# Reviewer report: Reviewer response for version 1

**DOI:** 10.3310/nihropenres.14975.r34348

**Published:** 2025-01-30

**Authors:** Welma Lubbe, Esther Oluwatoyin Famutimi

**Affiliations:** 1 North-West University, Potchefstroom, North West, South Africa; 2 NuMIQ, North-West University (Ringgold ID: 56405), Potchefstroom, North West, South Africa

Thank you for the opportunity to review this very important work. I applaud the authors for putting this manuscript together in such a comprehensive way. The comments made will improve the manuscript and may provide clarify on areas that make it more clear for the reader. It is not of a nature that require re-review, but I think would contribute to the quality of your report. 

 Topic:

 The topic is highly relevant given the continued high rates of neonatal mortality and the unprecedented global need to improve neonatal outcomes in low-resource settings.

 Considering the dynamism of the healthcare environment, the continuous nature of stakeholder engagement makes it an adaptive process, flexible, and responsive to changing needs and circumstances.

 Development area: 

 The term 'embedded' in the topic seems unnecessary.

 'The Learning to Harness Innovation in Global Health for Quality Care (HIGH-Q) programme' forms the basis of this paper and I wonder if HIGH-Q should not at least reflect in the title as well? e.g. 

 The HIGH-Q programme: A case study on engaging stakeholders in embedded newborn health services/systems research in Kenya - a continuous,  multiple actor process

 Abstract: 

 The focus is timely and significant for policymakers, healthcare providers, and researchers who aim to implement sustainable interventions despite the obvious decentralised health system.

 The abstract identifies the complexities of pooling multiple stakeholders together. This transparency and documentation make it more grounded and authenticates the realities of health system research.

 Development areas: 

 While the abstract mentions that engagement procedures vary from county to county, it skips over specific experiences learned.

 It will be appropriate to know the ratio of stakeholders involved in the study.

 Then, juggling the interests of these various stakeholders presented difficulties ranging from power dynamism to cultural or socio-economic differences.

 What stakeholder strategies were employed to manage the diverse interests and needs of stakeholders involved in the study? A bit more focused information on the actual stakeholder challenges and outcomes would be beneficial. The results seem to vague. 

 Add Kenya to the keywords

 Introduction: 

 The introduction has a clear definition and scope as it effectively defines stakeholder engagement and explains the various types of stakeholders.

 The introduction highlights important gaps in the literature, including the dearth of thorough explanations of stakeholder participation in real-world settings alongside the scant focus on the difficulties and conflicts involved in managing stakeholder input.

 Development areas: 

 The relationship between stakeholder participation and the HIGH-Q program needs a clearer explanation, as the current structure makes it difficult for readers to grasp the main issue.

 More background information about the HIGH-Q program, including its objectives, scope, and applicability to the Kenyan healthcare system, could be included in the introduction. HIGH-Q is only introduced in the study setting. It would be easier for readers to comprehend the relationship between the case study and the theoretical framework if this summary had been provided sooner. Or maybe ensure cross ref to box 1?

 Is the 'interventions' referred to the methods of the work packages? This seems a bit unclear. 

 Methods: 

 The methods highlight the principal investigators' proficiency with the Kenyan health system and give a concise summary of the stakeholder engagement group.

 Stakeholder mapping using Chevalier and Buckles' Rainbow diagram provides a methodical technique that successfully groups stakeholders according to their closeness to the research, impact, and interest.

 The engagement matrix analysis provides a structured understanding of stakeholder interests, concerns, and influence, enhancing the management of relationships.

 The conceptual framework used in this study allows for critical reflection on the ethical, strategic, and practical components of stakeholder involvement, which deepens the technique.

 Development areas: 

 The use of the Rainbow diagram for stakeholder mapping is effective, but providing a more detailed explanation of how stakeholders were assigned to specific strata would clarify and make the process more meaningful.

 The engagement matrix analysis is an appropriate tool for the study, but its development and use could be explained briefly.

 The actual application of the matrix might be made clearer, for example, by describing the precise categories that were employed and how stakeholders were assessed according to their influence, interest, and concerns.

 Stakeholder feedback is highlighted in this method, but it does not explain in depth how this input influenced the course of the study.

 The methods part does not address the difficulties or conflicts encountered during stakeholders’ engagement. This may be useful information for use in other contexts. It was also not very clear from the results discussion. 

 Taking care of issues like competing interests, opposition, or practical obstacles would provide a more accurate picture of the engagement process.

 Results: 

 High-Q is successfully placed into a broader framework of improving neonatal care in Kenya through the description of the NEST360 technology intervention and its connection to the Clinical Information Network (CIN). This emphasizes how the initiative helps improve care for ill and tiny babies throughout Africa.

 An excellent grasp of how the workforce intervention fits into the operational framework and local health systems is provided by the detailed explanation of the funding structure, managerial responsibilities, and the hiring of nurses and ward attendants.

 The work packages are well-organised, with clear research questions and methods for evaluating outcomes.

 The use of observations, interviews, and document reviews ensures a thorough, best-practice approach to data collection.

 The addition of precise information on sample sizes and achievements, such as "1,302 babies observed" and "38 mothers interviewed," adds credibility to the program and aids readers in evaluating its scope and advancement.

 The HIGH-Q programme's goal to influence neonatal care policy and practice is highly relevant.

 Development areas: 

 More clarification on how HIGH-Q’s workforce intervention aligns with NEST360’s technology goals and any overlap would strengthen understanding of their synergy.

 The rationale for adding three nurses and three ward assistants, including the evidence and reasoning behind these staffing levels, should be explained to clarify the intervention's design. 

 The governance structure of Kenya is briefly mentioned, but a deeper exploration of challenges from the devolved health system, including county-level variations in resources and priorities, would be helpful.

 A more thorough explanation of post-discharge care might be provided by outlining the program's plans to address present issues and the main gaps it seeks to close.

 Discussion:

 The strategic plan places a strong emphasis on collaboration for long-term value and wider effect throughout the research process while highlighting important goals including enhancing research quality, maintaining ethics, and optimizing policy impact.

 Development areas:

 The stakeholder mapping process mentions key groups, but more details on their roles, interactions, and contributions would clarify their impact on the research process.

 The strategy mentions stakeholder involvement but lacks details on challenges in engaging diverse groups across Kenya’s devolved healthcare system, limiting insights.

 Although the engagement approach is explained, little is known about how to assess its efficacy or how stakeholder input affected the design or results of the study.

 Stakeholder engagement objectives are described in this discussion. However, it is unclear how input affected the study topics, technique, or execution, which would have better illustrated the usefulness of engagement efforts.

 Although the program talks about involving stakeholders in the study, it would be helpful to include information about maintaining interactions once the project is over, such as participation in long-term follow-up or future research to fully measure the impact of the study.

 Conclusion:

 The conclusion recognises the difficulties presented by bureaucratic regulations, fluctuating power dynamics, and hierarchical systems, successfully highlighting the unexpected nature of stakeholder interaction.

 The conclusion emphasises the necessity for sufficient resources and reveals the significance of adaptability, responsiveness, and consistent effort in stakeholder involvement.

 Its emphasis on pragmatic factors for effective participation makes it a significant strength.

 Development areas:

 The conclusion lacks a clear, coherent flow and drifts between several subjects, including finance assistance, ethical approval, and involvement challenges. The reader may find it more difficult to understand the main points. Key 'take home messages' are lacking. 

 Although the conclusion highlights the value of stakeholder engagement, it does not explicitly show how it influenced the success or findings of the study.

 The argument would be more robust if there was more focus on how interaction affected the design, execution, or results of the research.

 This was a 'heavy' read, due to the complexity, but I think a very valuable contribution, especially in the field of intervention research aiming to change practice. Congratulations!

Is the rationale for developing the new method (or application) clearly explained?

Yes

Is the description of the method technically sound?

Yes

Are the conclusions about the method and its performance adequately supported by the findings presented in the article?

Yes

If any results are presented, are all the source data underlying the results available to ensure full reproducibility?

Yes

Are sufficient details provided to allow replication of the method development and its use by others?

Yes

Reviewer Expertise:

Neurodevelopmental supportive care. Health science implementation research.

We confirm that we have read this submission and believe that we have an appropriate level of expertise to confirm that it is of an acceptable scientific standard.

# Reviewer report: Reviewer response for version 1

**DOI:** 10.3310/nihropenres.14975.r34192

**Published:** 2025-01-17

**Authors:** Victoria J. Kain

**Affiliations:** 1 Griffith University, Brisbane, Australia

Thanks for opportunity to review - paper describes the stakeholder engagement process within the "Learning to Harness Innovation in Global Health for Quality Care (HIGH-Q)" program in Kenya, focusing on newborn care. The study aims to evaluate the impact of workforce interventions and technology integration in newborn units (NBUs) in four Kenyan hospitals. The authors detail the strategic framework, activities, and outcomes of stakeholder engagement, highlighting its moral, strategic, and practical dimensions. The study discusses the complexities and unpredictable nature of stakeholder engagement, emphasizing the need for continuous adaptation and adequate resources.

 While the paper provides a comprehensive description of the engagement process and activities, there is limited discussion on specific tools or templates used (e.g., stakeholder matrix design). Including appendices or supplementary materials with templates and engagement logs would enhance replicability.

 The authors mention that data is restricted due to institutional and legal constraints. While understandable, this limits reproducibility. A more detailed data-sharing plan or anonymized data samples could improve transparency.

 Needs to be some discussion on the variability in stakeholder engagement outcomes across the four counties, including factors influencing differences.

 How can this approach be adapted for larger-scale or multi-country studies?

 Your paper is very sound and provides valuable insights into stakeholder engagement in health systems research. However, addressing the replicability issues and providing additional context for the variability in engagement outcomes would significantly strengthen the paper as well as considering scalability.

Is the rationale for developing the new method (or application) clearly explained?

Yes

Is the description of the method technically sound?

Partly

Are the conclusions about the method and its performance adequately supported by the findings presented in the article?

Yes

If any results are presented, are all the source data underlying the results available to ensure full reproducibility?

Partly

Are sufficient details provided to allow replication of the method development and its use by others?

Partly

Reviewer Expertise:

Newborn care in lower resource setting.

I confirm that I have read this submission and believe that I have an appropriate level of expertise to confirm that it is of an acceptable scientific standard, however I have significant reservations, as outlined above.
